# siPools: highly complex but accurately defined siRNA pools eliminate off-target effects

**DOI:** 10.1093/nar/gku480

**Published:** 2014-05-28

**Authors:** Michael Hannus, Michaela Beitzinger, Julia C. Engelmann, Marie-Theresa Weickert, Rainer Spang, Stefan Hannus, Gunter Meister

**Affiliations:** 1 Biochemistry Center Regensburg (BZR), Laboratory for RNA Biology, University of Regensburg, Universitätsstrasse 31, 93053 Regensburg, Germany; 2 Intana Biosciences GmbH, Lochhamerstrasse 29A, 82152 Martinsried/Planegg, Germany; 3 siTools Biotech GmbH, Lochhamerstrasse 29A, 82152 Martinsried/Planegg, Germany; 4 Department of Statistical Bioinformatics, Institute for Functional Genomics, University of Regensburg, Josef-Engert-Straße 9, 93053 Regensburg, Germany

## Abstract

Short interfering RNAs (siRNAs) are widely used as tool for gene inactivation in basic research and therapeutic applications. One of the major shortcomings of siRNA experiments are sequence-specific off-target effects. Such effects are largely unpredictable because siRNAs can affect partially complementary sequences and function like microRNAs (miRNAs), which inhibit gene expression on mRNA stability or translational levels. Here we demonstrate that novel, enzymatically generated siRNA pools—referred to as siPools—containing up to 60 accurately defined siRNAs eliminate off-target effects. This is achieved by the low concentration of each individual siRNA diluting sequence-specific off-target effects below detection limits. In fact, whole transcriptome analyses reveal that single siRNA transfections can severely affect global gene expression. However, when complex siRNA pools are transfected, almost no transcriptome alterations are observed. Taken together, we present enzymatically produced complex but accurately defined siRNA pools with potent on-target silencing but without detectable off-target effects.

## INTRODUCTION

RNA interference (RNAi) is not only a potent cellular pathway to silence endogenous or exogenous genes but is also a widely used tool for sequence-specific gene knockdown (1). The trigger of RNAi is typically long double stranded (ds) RNA, which is processed to short interfering RNAs (siRNAs) by the cellular RNase III enzyme Dicer. SiRNAs are 21 nucleotides (nt) long dsRNAs, with 5′ phosphates and 2 nt 3′ overhangs (2,3). The strand that is complementary to the target RNAs is referred to as the guide or antisense strand, while the other strand is known as the passenger or sense strand. In further loading steps, which require the help of heat shock proteins, the guide strand is incorporated into the RNA-induced silencing complex (RISC) (4–6). The guide strand targets RISC to perfectly complementary target sites located on other RNAs leading to sequence-specific cleavage and further degradation of the target RNA (2).

The core protein component of RISC and direct interaction partner of siRNAs is a member of the Argonaute (Ago) protein family. Ago proteins are characterized by PAZ, MID and PIWI domains (7–9). The PAZ domain binds the 3′ end and the MID domain anchors the 5′ end of the siRNA. The PIWI domain is structurally similar to RNase H and indeed some Ago proteins possess endonuclease activity (10). These proteins are referred to as ‘slicers’ and facilitate the sequence-specific cleavage event in RNAi. The four human Argonaute proteins Ago1, Ago2, Ago3 and Ago4 are expressed in human somatic cells and interact with siRNAs (9,11). Although these proteins are very similar, only Ago2 is endonucleolytically active (12,13). Ago2 not only cleaves the complementary target RNA but uses its cleavage activity also during RISC loading: Ago2 binds to the ds siRNA and cleaves the passenger strand leading to faster and more efficient loading of the siRNA into Ago2-containing RISC complexes (14–16). Although less efficient, non-catalytic Ago proteins load siRNA guide strands in RNAi experiments (17,18) and it has been reported that non-catalytic Ago proteins can contribute to the knockdown as well (19).

After an initial hype and the hope that RNAi develops into a potent novel strategy for therapy, it became clear that several problems associated with RNAi experiments have not been solved. Besides delivery issues in RNAi-based therapeutic approaches, one of the major problems of siRNAs are off-target effects (20,21). Most siRNAs not only target their complementary on-targets but also an unknown number of off-targets. Off-target effects are sequence-specific and intrinsic to each siRNA molecule. Such effects mainly occur because of endogenous gene silencing pathways based on microRNAs (miRNAs) (22,23). MiRNAs are expressed from specific genes and inhibit the expression of target genes by binding to Ago proteins and hybridizing to only partially complementary target sites, which are typically located in the 3′ untranslated region (UTR) of target mRNAs (24). MiRNA-guided inhibition leads to translational silencing or deadenylation-induced mRNA decay (25). Nucleotides 2–8 of the miRNA comprise the ‘seed region’ and base pair perfectly with the target mRNA. The remaining nucleotides are less important for miRNA-guided targeting. Since siRNAs and miRNAs are indistinguishable within the cell, siRNAs can function like miRNAs and target mRNAs via their seed region. As the seed regions of siRNAs commonly match thousands of mRNAs, off-target effects are largely unpredictable. These effects are very common and vary from sequence to sequence. In addition, off-target effects can be caused by sequence-specific immune activation events or by a potential competition of siRNAs with endogenous miRNAs for effector proteins.

Here, we report the development of a new generation of siRNA tools without off-target effects. Using an enzymatic approach, we generate complex pools of accurately defined siRNAs. While synergistically silencing one single on-target gene, each individual siRNA is present at very low concentrations, effectively diluting off-target effects below detection limits. The novel enzymatic strategy allows for the free combination of individually selected siRNA sequences to obtain optimal siRNA pools. We refer to our novel siRNA pools as siPools and overcome off-target effects, the major shortcoming of classical siRNA reagents.

## MATERIALS AND METHODS

### Generation of complex siRNA pools

DNA templates were synthesized by GeneArt (Life Technologies). SiPool templates were *in vitro* transcribed using an integrated T7 promoter sequence followed by RNA precipitation with 2.5 M LiCl. Sense and antisense strands were annealed by incubation at 95°C for 5 min followed by slow cooling to room temperature. Annealed dsRNAs were subsequently digested with 10 U/μg RNase T1 to obtain ds siRNAs with 2 nt 3′ overhangs. RNase T1 digested siRNAs were purified by native 20% polyacrylamide gel electrophoresis and eluted from the gel overnight at 4°C in elution buffer (300 mM NaCl, 2 mM EDTA) followed by ethanol precipitation. To obtain siPools with 30 siRNAs, siPool #1 was mixed with siPool #2, and to generate siPool 45 with 45 different siRNAs, siPool #1 was mixed with siPool #2 and #3. siPool 60 containing 60 different siRNAs was mixed by the use of all four different siPools #1, #2, #3 and #4. TNRC6A, TNRC6B, TNRC6C and unspecific control siPools (neg. pools) each comprised of 30 individual siRNA sequences. For all template and siRNA sequences, see Supplementary Material. Ready-to-use siPools against various genes were purchased from siTools Biotech, Munich, Germany.

### siRNA sequences and low-complexity pool generation

Antisense strands of siRNA sequences were depicted: PolG siRNA off-T: 5′-AAUAUCCAGCGCUUCACCC-3′, Scyl1 siRNA off-T: 5′-ACAUUGUUGUGGAUGAGGC-3′; Mad2 siRNA: 5′-CCAAUCUUUCAGUUGUUCC3′; neg. ctrl. siRNA: 5′-UUGUCUUGCAUUCGACUAA-3′. esiRNAs (Sigma) were delivered as RNase III digested double-stranded products derived from the following sequences, PolG esiRNA: 5′-GGAAGAAGTGGGAGGTGGTTGCTGAACGGGCATGGAAGGGGGGCACAGAGTCAGAAATGTTCAATAAGCTTGAGAGCATTGCTACGTCTGACATACCACGTACCCCGGTGCTGGGCTGCTGCATCAGCCGAGCCCTGGAGCCCTCGGCTGTCCAGGAAGAGTTTATGACCAGCCGTGTGAATTGGGTGGTACAGAGCTCTGCTGTTGACTACTTACACCTCATGCTTGTGGCCATGAAGTGGCTGTTTGAAGAGT-3′, Scyl1 esiRNA: 5′-CAGCCGAGAAGCAAAAATTCTTCCAGGAGCTGAGCAAGAGCCTGGACGCATTCCCTGAGGATTTCTGTCGGCACAAGGTGCTGCCCCAGCTGCTGACCGCCTTCGAGTTCGGCAATGCTGGGGCCGTTGTCCTCACGCCCCTCTTCAAGGTGGGCAAGTTCCTGAGCGCTGAGGAGTATCAGCAGAAGATCATCCCTGTGGTGGTCAAGATGTTCTCATCCACTGACCGGGCCATGCGCATCCGCCTCCTGCAGCAGATGGAGCAGTTCATCCAGTACCTTGACGAGCCAACAGTCAACACCCAGATCTTCCCCCACGTCGTACATGGCTTCCTGGACACCAACCCTGCCATCCGGGAGCAGACGGTCAAGTCCATGCTGCTCCTGGCCCCAAAGCTGAACGAGGCCAACCTCAATGTGGAGCTGA-3′.

To generate low-complexity pools, the following siRNAs were used: PolG siRNA 1: 5′-UCAUCCGACAGCCGAUACC-3′, PolG siRNA 2: 5′-AAUUCUUGCAGGUCCCACU-3′, PolG siRNA 3: 5′-GCUAUUACCAUCCUUGUGA-3′, PolG siRNA 4: 5′-UUAAACUGCAUUAGUAAGC-3′. Scyl1 siRNA 1: 5′-UUUCUCAGGAUCUACAGUGAG-3′, Scyl1 siRNA 2: 5′-UUGAGGUAUAUUCCCAACGGG-3′, Scyl1 siRNA 3: 5′-UUGGUUUCUACAAAGCGGUUG-3′, Scyl1 siRNA 4: 5′-UUGUACAAUAAAUACAUCUGU-3′. Three siRNAs were mixed in different combinations with the corresponding siRNA off-T to obtain four different siRNA-containing low-complexity pools with possible Mad2 off-target effects. For pool #1, siRNA 1, 2, 3; for pool #2, siRNA 1, 2, 4; and for pool #3, siRNA 1, 3 and 4 were mixed with off-T siRNAs. Low-complexity pools consisting of siRNA 1–4 were used as non-Mad2 off-target controls (pool #4).

### Cell culture and transfections

HeLa cells were cultivated in Dulbecco's modified Eagle's medium substituted with 10% FCS (fetal calf serum) and penicillin/streptomycin. siRNA transfections were done using Lipofectamine RNAiMax (Life Technologies) according to the manufacturer's protocol. Cells were harvested 24 h or 48 h after transfection.

### qPCR and western blot

RNA was isolated 24 h after transfection followed by cDNA synthesis and quantitative polymerase chain reaction (qPCR). Following primers were used: PolG for: 5′-TTCCAGGACCTGATGCAGTA-3′, PolG rev: 5′- ACAGGCAGGTAGGAGACACC-3′, Scyl1 for: 5′-CTGGAGGAAGTGGAGAAGGA-3′, Scyl1 rev: 5′-TCAGCTTGGAGGTGAGTGAG-3′, Mad2 for: 5′-AGATGACAGTGCACCCAGAG-3′, Mad2 rev: 5′-TCCAACAGTGGCAGAAATGT-3′ GAPDH for: 5′-ATGGGTGTGAACCATGAGAA-3′, GAPDH rev: 5′-GTGCTAAGCAGTTGGTGGTG-3′, TNRC6A for: 5′-CCCTCAGGTTCCAGTTTCAT-3′, TNRC6A rev: 5′-GCTGGTTTAGCTGGGATAGC-3′, TNRC6B for: 5′-ATGGTTCTGGCTTCAGCTCT-3′, TNRC6B rev: 5′-CATATTGGCTTCCTGTGTGG-3′, TNRC6C for: 5′-TAGCAAGCATGGTGCTATCC-3′, TNRC6C rev: 5′-GTACCGGCCATAGGAGTCAT-3′, OAS1 for: 5′-TGCCATTGACATCATCTGTG-3′, OAS1 rev: 5′-GAGCCACCCTTTACCACCT-3′, INFB1 for: 5′-AGTAGGCGACACTGTTCGTG-3′, IFNB1 rev: 5′-GAAGCACAACAGGAGAGCAA-3′, IL6 for: 5′-AATGAGGAGACTTGCCTGGT-3′, IL6 rev: 5′-GCAGGAACTGGATCAGGACT-3′, STAT1 for: 5′-AATTGACCTCGAGACGACCT-3′, STAT1 rev: 5′-CACATGGTGGAGTCAGGAAG-3′.

For western blot analysis cells were harvested and lysed in NET buffer (50 mM Tris pH 7.4, 150 mM NaCl, 5 mM EDTA, 0.5% NP40, 10% glycerol) 48 h after transfection. Proteins were separated via sodium dodecyl sulphate-polyacrylamide gel electrophoresis followed by semi-dry electroblotting. Following antibodies were used: polyclonal anti-MAD2 (Bethyl Laboratories) at a dilution of 1:5000 and a monoclonal mouse anti beta actin antibody (clone AC15 from Abcam) at a dilution of 1:5000 in TBS-Tween with 5% milk powder. Fluorescently labeled IRDye 800 CW antibodies were used as secondary antibodies (Li-COR). Western blots were imaged with an Odyssey Fluorescence scanner (Li-COR).

### Dual luciferase assay

To generate an off-target reporter construct, a modified pMIR dual luciferase reporter plasmid (26) was used. The 3′-UTR of Mad2 was amplified by PCR and cloned into the corresponding SacI and PmeI sites of pMIR. Following primers were used: Mad2-for 5′-GATCGAGCTCGGATGACATGAGGAAAATAA-3′ and Mad2-rev 5′-GATCGTTTAAACAAGACAAATTTAAAACAAACTTA-3′. To analyze the role of TNRC6 family member knockdown, luciferase assays were performed using pmir-RL-TK with HMGA2 3′-UTR and a HMGA2 3′-UTR with mutated let-7 miRNA binding sites (27). As positive control, 2′OMe inhibitors targeting let-7a miRNA (r[2′OMe](AACTATACAACCTACTACCTCA)dT) or a scrambled sequence (r[2′-OMe](GCCAAGUGUUAAAGGCCAAACUACGUUGAGAG)dT) were used. HeLa cells were transfected in 96 well plates with 1, 3 or 10 nM siRNAs and 20 ng 3′-UTR plasmid using Lipofectamine 2000 (Life Technologies). Cells were harvested and lysed in passive lysis buffer (Promega) 24 h after transfection. Firefly/Renilla luminescence ratios were normalized to corresponding ratios of the empty pMIR plasmid or to HMGA2 mutant vector.

### Co-immunoprecipitation and northern blotting

HeLa cells were transfected with 10 nM siPools and lysed in NET buffer (50 mM Tris pH 7.4, 150 mM NaCl, 5 mM EDTA, 0.5% NP40, 10% glycerol) 48 h after transfection. Lysates were used for Ago2-siRNA co-immunoprecipitation. Protein-G sepharose beads (GE) were pre-incubated with monoclonal anti-Ago2 (11A9) antibody (28). Lysates were incubated with the Ago2 antibody-coupled beads for 4 h at 4°C. Immunoprecipitations were subsequently washed with NET buffer followed by proteinase K digestion and phenol/chloroform extraction of bound RNAs. Northern blot was performed as described earlier (29). As probes for siRNA detection, antisense DNA oligos for the corresponding off-T siRNAs were used: PolG Pool #1 siRNA off-T guide: 5′-GGGTGAAGCGCTGGATATT-3′, PolG Pool #1 siRNA off-T passenger: 5′-AATATCCAGCGCTTCACCC-3′, Scyl1 Pool #1 siRNA off-T guide: 5′-GCCTCATCCACAACAATGT-3′, Scyl1 Pool #1 siRNA off-T passenger: 5′-ACATTGTTGTGGATGAGGC-3′.

### Transcriptome-wide gene expression analysis

HeLa cells were transfected with 3 nM Scyl1 siRNA, Scyl1 siPool 15, Scyl1 siPool 60, or mock transfected in three biological replicates. RNA was isolated 48 h after transfection and further processed for Affymetrix Human Gene 1.0 ST array analysis. The Human Gene 1.0 ST array platform from Affymetrix (Santa Clara, CA, USA) was used to assess transcriptome-wide expression profiles. Normalization of raw intensity values from CEL files was performed using variance stabilization (30), and the median polish was used to summarize individual probes to an expression level per transcript. Transcripts were defined with a custom chip description file based on Ensembl transcript identifiers (31).

The normalized data on transcript level allow for distinguishing between transcripts with different 3′-UTRs of the same gene. From the 134 429 different transcripts only the transcripts annotated as ‘protein-coding’ in the Ensembl genes database, version 69, were retained. This yielded 78 622 transcripts. Non- and low-expressed transcripts were then filtered out before testing for differential expression by requiring at least two expression values of the 12 samples of the data sets to be above the 40th percentile of all expression values. Differential transcript expression between cells treated with one or more siRNAs and untreated cells was estimated using *limma* (32). Because a large number of tests were performed for differential expression, false positive findings were controlled with the false discovery rate (FDR). Instead of multiple testing adjusted *P*-values, so-called *q*-values are reported which indicate the largest FDR at which the transcript could be considered significant. Transcripts with a *q*-value below 10^−6^ were considered significant differentially expressed.

MCF7 cells were transfected with 3 nM Scyl1 siPool 60, Scyl1 esiRNA (Sigma), or mock transfected in three biological replicates. RNA was isolated 48 h after transfection. Transcription profiles were analyzed using the Human Gene 2.0 ST array platform (Affymetrix). Raw intensity values (from CEL files) were normalized using RMA (*oligo* (33)). The nsFilter method from *genefilter* was used to remove the 50% of features with lowest variability. Probe-to-gene mappings were taken from *hugene20sttranscriptcluster.db*. Probes not mapping to an NCBI Gene ID were removed from the analysis, leaving 9518 genes for differential expression analysis. Differentially expressed genes were determined using *limma*, with a *q*-value cutoff of 10^−3^. GO over-representation analysis of differentially expressed genes was performed using HTSanalyzeR (34). All log_2_ fold changes reported are in the form of siRNA experiment versus control.

Analyses were performed within the statistical programming environment R and using Bioconductor (35) packages.

### Sequence analysis

Human 3′-UTR sequences were retrieved from Ensembl version 68 for the transcripts represented on the cartridge microarray. The reverse compliment of the siRNA seed sequences (7 nt) was searched for in the 3′-UTRs of the transcripts and 2292 transcripts with a seed sequence match were found. To analyze whether there is significant enrichment of transcripts with a seed-binding site among the repressed transcripts after siRNA transfection, the number of repressed transcripts with a seed match was compared to the mean number of transcripts with a random seed match. Seven thousand five hundred random seed sequences were drawn and from these, 2000 with similar numbers of transcripts with a seed match in the 3′-UTR (range 1692–2892 transcripts per sequence) were drawn and the number of repressed transcripts with a seed match were recorded for each random seed sequence. The distribution of these values was used to calculate the *P*-value for the number of repressed transcripts with a seed match for the Scyl1 siRNA to belong to the same distribution. For the pool 15 and pool 60, three additional siRNA seed sequences were drawn from the pools and tested analogously.

## RESULTS

### Enzymatic production of highly complex siRNA pools

We hypothesized that siRNA pools containing up to 60 individual siRNAs against one target possess very low off-target effects because of the very low concentration of each individual siRNA. However, chemical synthesis of high numbers of siRNAs is cost-intensive and therefore not practicable. Therefore, we set out to generate complex siRNA pools enzymatically. For our experiments, we chose the human target genes PolG and Scyl1. siRNAs with strong off-target effects against the gene MAD2 had been reported for both genes (36). These siRNAs represent ideal test cases for further downstream experiments on off-target effects (see below).

**Figure 1.**
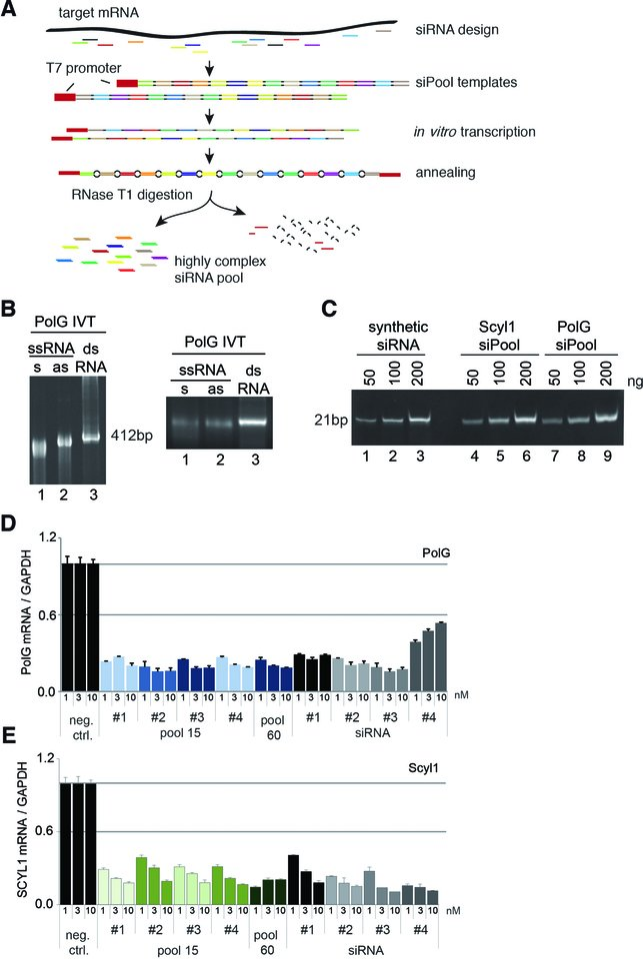
siPool generation and on-target activity. (**A**) Schematic overview of siPool construction. SiRNA sequences are designed and arranged in tandem separated by specific spacer sequences. siPool transcripts are *in vitro* transcribed by T7 polymerase and annealed. The resulting siPool precursors contain complementary siRNA sequences flanked by single-stranded spacer regions. The spacer sequences allow RNase T1 digestion resulting in double-stranded siRNAs with 2 nt 3′ overhangs. (**B**) *In vitro* transcription and annealing of siPools. 600 ng (left) or 800 ng (right) *in vitro* transcribed (IVT) sense and antisense strands (lanes 1 and 2) were annealed to a double-stranded siPool precursor (lane 3), loaded onto a 5% native polyacrylamide gel (left) or on a 1% native agarose gel (right). (**C**) Comparison of purified siPools with a synthetic siRNA. 50, 100 or 200 ng of purified siPools (lanes 4–9) or a synthetic siRNA (lanes 1–3) was loaded onto a 20% native polyacrylamide gel and visualized by ethidium bromide staining. (**D** and **E**) HeLa cells were transfected with 1, 3 or 10 nM concentrations of siPools containing 15 siRNAs (pool 15 #1–4), a combination of all 15 siRNA-siPools resulting in a siPool containing 60 different siRNAs (pool 60) or specific siRNAs (#1–4) directed against PolG (D) or Scyl1 (E).

For siRNA pool production, we selected siRNA sequences using standard siRNA prediction tools, which are connected by non-complementary linker sequences (Figure 1A). The DNA template containing the siRNA and linker sequences contain T7 promoters to allow for the transcription of two RNA strands, which are complementary over the siRNA sequences but not the linkers. G nucleotides placed at appropriate positions within the linker regions allow for specific digestion with the endonuclease T1, an enzyme that cleaves after G only in single-stranded RNA. T1 treatment results in siRNAs with 2 nt 3′ overhangs (Figure 1A).

The two strands were transcribed, annealed and analyzed on a native polyacrylamide (PAA) gel (Figure 1B, left panel) or a native agarose gel (right panel). In all cases, distinct bands were observed that were readily annealed to the bulged dsRNA as evident from a size shift and enhanced ethidium bromide staining intensity.

The ds siRNA precursors were now incubated with the endonuclease T1 (Figure 1C). T1 treatment resulted in a complete disappearance of the precursor and the appearance of a distinct, 21 nt long cleavage product, which was further purified by gel purification. Our siPool production procedure combining *in vitro* transcription and T1 digestion produces siRNAs that are not distinguishable from commercially available synthetic siRNAs (Figure 1C).

### Complex siRNA pools show highly efficient on-target activity

In order to demonstrate the functionality of siPools, we generated four different pools against PolG (Figure 1D) or Scyl1 (Figure 1E), each containing an individually defined set of 15 siRNA sequences (pool #1–4). The individual as well as the combined pools (pool 60) were transfected into HeLa cells in three different concentrations and the knockdown was measured on the mRNA level by qPCR. In addition, four synthetic siRNAs against PolG or Scyl1 were transfected using the same concentrations. At all concentrations used, the siRNA pools as well as the individual synthetic siRNAs against PolG or Scyl1 show similar on-target activity. This is also observed when IC50 values are determined (Supplementary Figure S1). The synthetic siRNA #4 against PolG, however, appears to be less efficient. Thus, our enzymatically produced complex siRNA pools are as efficient as synthetic siRNAs and can be broadly used for gene knockdown experiments.

### Simultaneous knockdown of redundant gene family members

**Figure 2.**
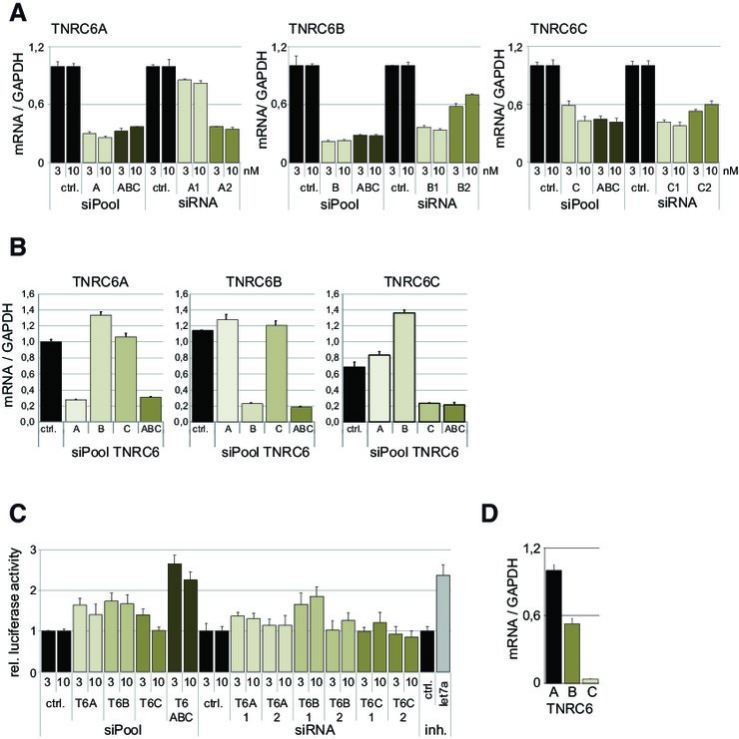
siPools efficiently knock down redundant gene family members. (**A**) HeLa cells were transfected with 3 or 10 nM concentrations of siPools targeting TNRC6A (‘A’, left panel), TNRC6B (‘B’, middle panel), TNRC6C (‘C’, right panel) or a combination of all three siPools (‘ABC’). Specific siRNAs against TNRC6A, TNRC6B or TNRC6C were transfected in similar concentrations. As a negative control for TNRC6 targeting siPools, an unspecific control siPool, and for siRNAs an unspecific control siRNA, was used. mRNA levels were measured by qPCR and normalized to GAPDH, and relative expression levels were calculated based on transfection of an unspecific control siPool or an unspecific control siRNA (ctrl.). (**B**) siPools specifically knock down individual family members. HeLa cells were transfected with 3 nM concentrations of siPools targeting TNRC6A (‘A’), TNRC6B (‘B’), TNRC6C (‘C’) or a combination of all three siPools (‘ABC’). mRNA levels of TNRC6A (left panel), TNRC6B (middle panel) or TNRC6C (right panel) were measured by qPCR and normalized to GAPDH. Relative expression levels were calculated based on transfection of an unspecific control siPool or an unspecific control siRNA (ctrl.). (**C**) Dual luciferase assay: HeLa cells were co-transfected with HMGA2 3′-UTR dual luciferase expression vector and with 3 or 10 nM siRNAs, or siPools targeting TNRC6A (T6A), TNRC6B (T6B), TNRC6C (T6C) or a combination of all siPools targeting TNRC6A, TNRC6B and TNRC6C (T6 ABC). As positive control we used a let-7a 2′OMe miRNA inhibitor which was normalized to an unspecific control 2′OMe inhibitor (ctrl.). Relative luciferase activity was calculated using the ratio for firefly/Renilla luciferase and via normalization to the corresponding ratios of an HMGA2 3′-UTR with mutated let-7a binding sites. All ratios were further normalized to a corresponding unspecific negative control (ctrl.) siPool or siRNA. (**D**) qPCR analysis of TNRC6A, TNRC6B and TNRC6C expression levels relative to GAPDH in HeLa cells.

The convenient production procedure as well as the efficient knockdown prompted us to ask whether we can knock down redundant gene family members using a single siPool. For our analysis, we chose the human TNRC6 protein family comprising TNRC6A, TNRC6B and TNRC6C. These proteins are downstream factors of Ago proteins and are essential for miRNA-guided gene silencing (25). We generated siPools against the individual TNRC6 proteins (Figure 2A, siPool A, B and C) and also combined them to one siPool (siPool ABC). All siPools knocked down their individual on-targets without affecting the mRNA levels of the other TNRC6 gene (Figure 2A and B). Single siRNAs knocked down the TNRC6 genes as well but showed rather variable efficiencies (Figure 2A). Strikingly, the siPool ABC targeting all TNRC6 genes indeed reduced the mRNA levels of each family member efficiently (Figure 2A). We next tested the consequences of TNRC6 gene knockdown on endogenous miRNA function using miRNA reporter assays based on a luciferase gene controlled by the HMGA2 3′-UTR (Figure 2C). This 3′-UTR contains seven let-7a binding sites and is repressed by the miRNA machinery (37). Inhibition of let-7a by antisense inhibitors leads to a relief of repression (gray bar) indicating that the reporter system is indeed under the control of let-7a. Since TNRC6 proteins are mediators of miRNA-guided gene silencing, we hypothesized that knockdown of these proteins should lead to a block of miRNA-guided repression and thus increased luciferase signals. Consistently, knockdown of TNRC6A or B either by the siPools or by individual siRNAs relieved repression and led to an increase of luciferase activity of about 1.5–1.8-fold (Figure 2C). Knockdown of TNRC6C alone had only a minor effect on luciferase activity. Of note, TNRC6C expression is much weaker in the HeLa cells that have been used and this might explain the stronger variation of the TNRC6C measurements (Figure 2A, B and D). Simultaneous knockdown of all three family members by the siPool ABC released repression by 2.3- to 2.5-fold, a similar range observed by the antisense inhibitor against let-7a. Thus, an increased effect compared to the single knockdowns indicates that the three human proteins function redundantly on the HMGA2 reporter. In summary, we demonstrate that siPools can be used for efficient knockdown of redundant gene family members and, furthermore, the siPool ABC against all TNRC6 genes is a valuable control tool for experiments aiming at identification of miRNA target genes.

### Complex siRNA pools do not cause measurable off-target effects

**Figure 3.**
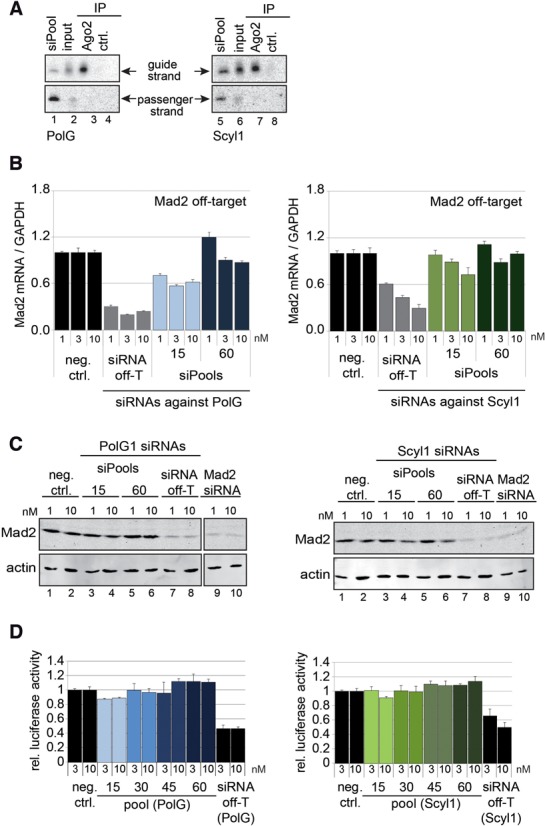
Off-target activity of different siPools. (**A**) HeLa cells were transfected with 10 nM siPool 60. To validate that the specific off-T siRNAs are present in the pools, Ago2 was immunoprecipitated from the lysates and passenger and guide strands of PolG off-T (left) or Scyl1 off-T (right) siRNAs were analyzed by northern blotting. As positive controls, 3 pmol of total siPools and 2.5% input material were used. (**B**) HeLa cells were transfected with 1, 3 or 10 nM siPool 15 #1, siPool 60 or specific off-T siRNAs directed against PolG (blue) or Scyl1 (green). Mad2 mRNA levels were measured by qPCR and normalized to GAPDH. Relative expression levels were calculated based on transfection of an unspecific control siRNA (neg. ctrl.). (**C**) Experiment was performed as described in (B). MAD2 protein levels were analyzed by western blotting 48 h after transfection. A specific MAD2 siRNA served as a positive control (lanes 9 and 10). Actin expression levels were used as loading controls (lower panels). (**D**) HeLa cells were transfected with 3 or 10 nM siRNA off-T or siPools containing 15, 30, 45 or 60 different siRNAs directed against PolG (blue) or Scyl1 (green). Off-target activity was analyzed using MAD2 3′-UTR controlling firefly-luciferase activity. Relative luciferase activity was calculated using the ratio of firefly/Renilla luciferase and via normalization to the corresponding ratios of the empty control vector.

To directly test our hypothesis that off-target effects of individual siRNAs are eliminated in complex siRNA pools, we took advantages of the known off-target effect against MAD2 of the abovementioned siRNAs against PolG or Scyl1 (36). To ensure that the off-target siRNAs were indeed present in our pools, we transfected the 60 siRNA-containing pools against PolG or Scyl1 into HeLa cells and immunoprecipitated Ago2 from the cell lysates (Figure 3A). The off-target siRNAs were analyzed by northern blotting using probes against the guide (upper panel) or the passenger strand (lower panel). The guide strand was readily detectable in Ago2 complexes indicating that siRNAs are efficiently processed and loaded and our siPools can be used for further off-target analysis.

Next, we reproduced the described Mad2 off-target effect (Figure 3B). Consistent with the published literature, both the siRNA against PolG (siRNA off-T, left panel) and the siRNA against Scyl1 (siRNA off-T, right panel) strongly reduced Mad2 mRNA levels when transfected into HeLa cells. We next placed the same siRNAs into a siRNA pool containing 14 (siPool 15) or 59 (siPool 60) additional siRNAs (Figure 3B, blue and green columns). The pools were transfected into HeLa cells and MAD2 mRNA levels were measured by qPCR. Strikingly, the MAD2 off-target effect of the two siRNAs was markedly reduced when 15 siRNAs were combined and hardly measurable when 60 siRNAs were used. To further solidify our results, we analyzed Mad2 protein reduction by PolG and Scyl1 siRNA off-target effects (Figure 3C). HeLa cells were transfected with siRNAs against PolG (left panel: siPools 15 and 60, single siRNA off-T) or Scyl1 (right panel: siPools 15 and 60, single siRNA off-T). Cells were lysed and protein extracts were analyzed by western blotting against MAD2. In both cases, a siRNA directed against MAD2 was added for further specificity control. In accordance with our results on MAD2 mRNA levels, we find that both off-target siRNAs as well as the control siRNA directed against MAD2 strongly reduce MAD2 protein levels (left and right panels, lanes 7–10). However, when the same off-target siRNAs are placed in complex siRNA pools, the MAD2 protein depletion is strongly reduced (left and right panels, lanes 3–6).

### Off-target effects are lost in a luciferase reporter system

To further solidify our data, we generated luciferase reporters containing miRNA-like binding sites for the PolG off-target siRNA or the Scyl1 off-target siRNA (Figure 3D). The single off-target siRNAs were transfected into HeLa cells and a reduction of the luciferase activity was observed (left and right panels, siRNA off-T). However, when the siRNAs were part of complex siRNA pools, the reduction of the luciferase activity was abolished. Furthermore, we analyzed the complexity requirements of the pools for off-target elimination. While the Scyl1 off-target effect was already eliminated when 15 siRNAs were used, the effects of the PolG pools were slightly stronger in pools with higher complexity (left panel, compare pool 15 with pool 30), suggesting that approximately 30 siRNAs within a pool are sufficient for off-target elimination. In summary, using independent approaches we demonstrate that off-target effects of individual siRNAs can be strongly reduced by combining multiple siRNAs to complex pools.

### siPools affect global transcript levels less than single siRNAs

**Figure 4.**
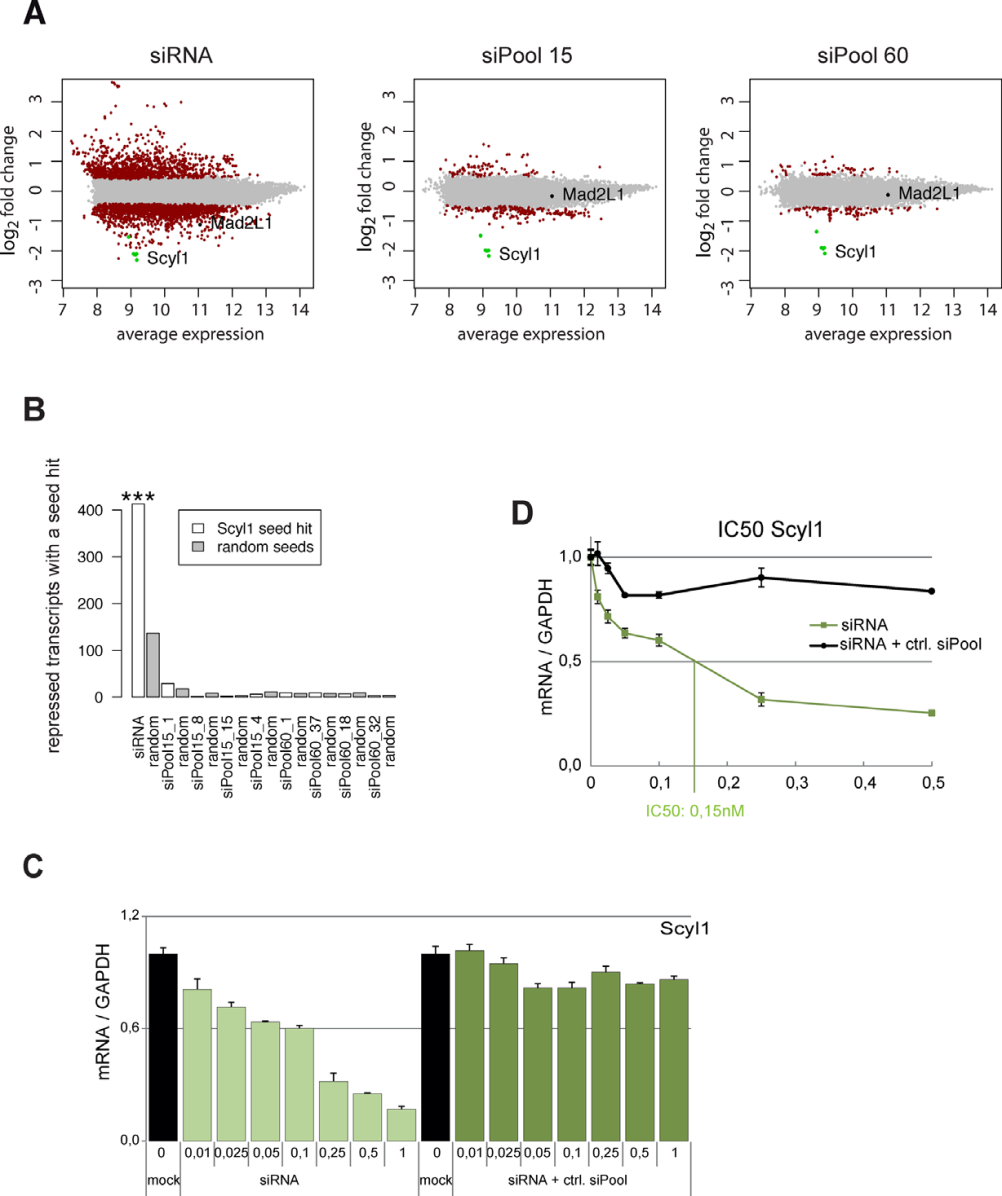
Global mRNA expression profiles upon siRNA transfection. HeLa cells were transfected with 3 nM of a Scyl1-targeting siRNA and siPools. (**A**) Differential mRNA expression in the single siRNA, pool of 15 sequences and pool of 60 sequences experiment. The horizontal axis shows the average expression level over siRNA-treated and control experiments, and the vertical axis shows the difference in expression between treated and control samples (log_2_ fold change). Transcripts differentially expressed at a *q*-value of 10^−6^ are highlighted in red, and all others are shown in gray. Scyl1 transcripts are highlighted in green, Mad2 (isoform MAD2L1 was targeted) in black. The siRNA experiment induces more genes to change expression levels than the pool experiments. The off-target MAD2L1 is significant differentially expressed in the siRNA experiment, but not in the pool experiments. (**B**) Enrichment of seed complementary sites in the 3′-UTRs of repressed transcripts. White bars show the number of repressed transcripts with a Scyl1 siRNA complementary seed sequence in the single siRNA (siRNA) and the pool of 15 (pool 15, bar labeled siPool15_1) and 60 sequences (pool 60, bar labeled siPool60_1). Gray bars show the mean number of repressed transcripts in the respective experiment with a complementary site to a random seed sequence. The enrichment in the single siRNA transfection experiment is significant with *P* << 0.001, while the same sequence as well as three additional randomly chosen sequences of the pools are not significantly enriched in the repressed transcripts. (**C** and **D**) Activity of single siRNAs as part of negative control siPools. HeLa cells were transfected with indicated concentrations in nM of Scyl off-T siRNA alone or mixed in an unspecific ctrl. siPool to an equal concentration of individual siRNAs of the siPool. IC50 values are depicted in the line graph (C); in addition, data are also shown in a bar graph (D). mRNA levels were measured via qPCR and normalized to GAPDH.

We next investigated effects of single siRNAs or siPools on global mRNA levels using microarrays (Figure 4A and B). The Scyl1 siRNA with off-target against MAD2 or siPools containing 15 or 60 siRNAs were transfected into HeLa cells and global mRNA changes were analyzed. Mock transfection served as control. The single siRNA against Scyl1 reduced the levels of all Scyl1 transcripts efficiently (Figure 4A, left panel, green circles). However, MAD2 and many other transcripts were also strongly reduced. When siPools containing 15 (middle panel) or 60 (right panel) siRNAs were used, Scyl1 transcripts were efficiently knocked down as well but Mad2 remained unaffected. Furthermore, global transcript levels were affected only mildly when 15 siRNAs were used and almost no alterations were observed when the 60 siRNA siPool was transfected. Focusing on the repressed transcripts after single siRNA or siPool transfection, transcripts with a Scyl1 siRNA complementary seed sequence in the 3′-UTR are significantly enriched in the single siRNA transfection experiment (*P* = 3 × 10^−16^), but not in the siPool transfections (Figure 4B). Therefore, our siPool strategy not only depletes the on-target transcripts very efficiently, but also perturbs global transcript levels only marginally.

It is conceivable that a single siRNA within the pool contributes strongly to the on-target activity. Thus, a potent siRNA could simply be diluted with a standard off-target pool to reduce off-target effects. To test this directly, we mixed a potent siRNA against Scyl1 with an unrelated control siPool and compared the on-target activity of the pool with the activity of the single siRNA (Figure 4C). We clearly observe that a simple dilution of a potent siRNA with unrelated siRNAs causes a dramatic loss of on-target activity. This is also observed when IC50 values are determined (Figure 4D). As expected, off-target effects are strongly reduced by siRNA dilution (Supplementary Figure S2). Thus, all siRNAs within a complex pool contribute to on-target activity and complex siPools against one mRNA target overcome off-target effects without the loss of on-target activity.

### Comparison of complex siRNA pools with commonly used siRNA reagents

**Figure 5.**
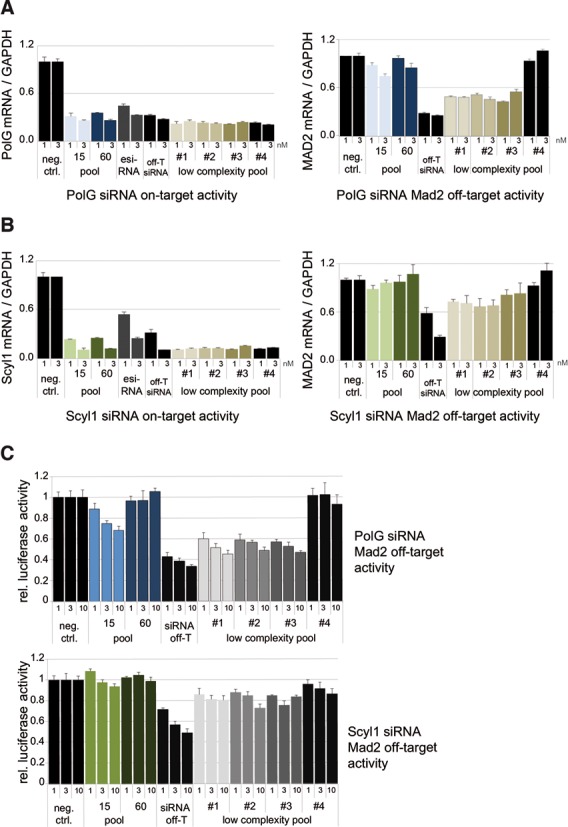
Comparison of siPools with other available RNAi reagents. qPCR analysis of on- (left panels in (A) and (B)) and off-target (right panels in (A) and (B)) activities of various siRNA tools. HeLa cells were transfected with 1 or 3 nM siPool 15, siPool 60, single off-T siRNA, esiRNAs and four different low-complexity pools directed against PolG (**A**) or Scyl1 (**B**). mRNA levels were normalized to GAPDH and relative expression levels were calculated using a negative control siRNA. Low-complexity pool #4 served as a MAD2 off-target negative control. (**C**) HeLa cells were transfected with 1, 3 or 10 nM siRNA off-T, siPools with 15 or 60 different siRNAs and four different low-complexity pools directed against PolG (blue) or Scyl1 (green). Off-target activity was analyzed using a reporter system based on firefly-luciferase activity controlled by the MAD2 3′-UTR. Relative luciferase activity was calculated using the ratio of firefly/Renilla luciferase and via normalization to the corresponding ratios of the empty control vector. Low-complexity pool #4 served as a MAD2 off-target negative control.

Various RNAi reagents are available including synthetic single siRNAs, RNase III generated siRNAs from long dsRNA precursors (esiRNAs) (38) or siRNA pools with low complexity containing only four synthetic siRNAs (smart pools). To evaluate our siPools with respect to other RNAi tools, we compared on- and off-target effects of commercially available RNAi reagents with our siPools (Figure 5). We first tested PolG on-target activities (Figure 5A, upper panel). A single siRNA (off-T siRNA; MAD2 off-target), an esiRNA pool, four low-complexity pools (#1–3 contain the off-T siRNA) as well as siPools with 15 or 60 siRNAs were transfected and PolG mRNA levels were measured using qPCR. As expected, all reagents show similar PolG knockdown efficiency. When MAD2 mRNA levels were analyzed, the off-target siRNA efficiently silenced the Mad2 mRNA as well (lower panel). Also, when placing the off-target siRNA into low-complexity pools (#1–3), the off-target activity was slightly reduced but still readily detectable. In agreement with our previous results, our 15 or 60 siRNAs containing siPools eliminated the MAD2 off-target effect. Of note, low-complexity pool #4 does not contain the off-target siRNA and serves as control in this experiment. esiRNAs, which resemble a random mixture of short dsRNA fragments from a long double-stranded precursor RNA, do not contain the specific Mad2 off-T siRNA and were therefore not included. Similar results were obtained when the Scyl1 off-target siRNA was used (Figure 5B). Finally, we utilized the abovementioned luciferase reporter system carrying a miRNA-like binding site for the PolG and Scyl1 off-target siRNAs (Figure 5C, upper and lower panels). Consistent with the mRNA data, our siPools eliminated off-target effects in this assay, while low-complexity pools (#1–3) led only to a moderate reduction. Taken together, our data demonstrate that while on-target silencing of siPools matches the efficiency of commercially available single siRNAs or low-complexity pools, only siPools eliminated off-target effects in these experiments.

### siPools do not cause measurable interferon responses

**Figure 6.**
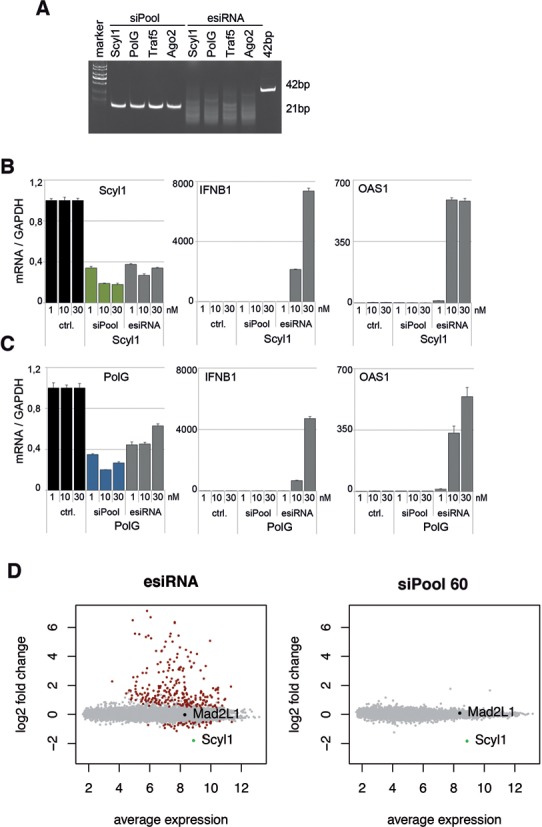
Analysis of interferon response induction. (**A**) 400 ng of esiRNAs or 200 ng siRNA pools both against Scyl1, PolG, Traf5 and Ago2 were loaded onto a 20% native PAA gel and stained with ethidium bromide. (**B** and **C**) MCF7 cells were transfected with 1, 10 or 30 nM siPools or corresponding esiRNAs against Scyl1 (B) or PolG (C). mRNA levels were normalized to GAPDH and relative expression levels were calculated using a negative control siPool. (**D**) MCF7 cells were transfected with 10 nM esiRNAs (left) or a siPool containing 60 siRNAs (right) against Scyl1. mRNA expression profiles were assessed using microarray.

Since siPools and esiRNAs derive from longer dsRNA precursors and such precursors might cause interferon response, we tested the expression of interferon response genes after siRNA transfection (Figure 6). siPools against four different targets (PolG, Scyl1, Traf5 and Ago2) were compared to the corresponding commercial esiRNAs obtained from Sigma. While siPools show distinct 21 nt long bands, all purchased esiRNAs were characterized by an RNA smear ranging from 15 to more than 40 nt (Figure 6A). For interferon response experiments, we changed to MCF7 cells, as HeLa cells are known to be rather insensitive to interferon inducing agents. All four target genes were efficiently knocked down by the siPools, while the esiRNA-mediated knockdown was slightly less efficient (Figure 6B and C, left panels, and Supplementary Figure S3). We next analyzed the expression of the interferon response genes IFNB1 and OAS1 upon knockdown. While siPools did not cause expression of IFNB1 or OAS1, esiRNAs led to a strong (Scyl1, PolG) or to a medium to low (Traf5, Ago2) interferon response (Figure 3B and C and Supplementary Figure S3). Similar results were obtained when the interferon response genes IL6 and STAT1 were measured (Supplementary Figure S4). For a broader overview, we analyzed global mRNA changes upon esiRNA or siPool transfection in MCF7 cells (Figure 6D). While transfection of a siPool containing 60 siRNAs did not lead to obvious mRNA changes except for the on-target Scyl1 (right panel), the esiRNA triggered severe alterations in transcript levels (left panel). Interestingly, a large part of the up-regulated mRNAs originates from interferon response genes (Supplementary Table S1). Together, our data suggest that esiRNAs can trigger the cellular interferon response due to the longer dsRNA fragments they contain. This is not observed for siPools.

## DISCUSSION

Off-target effects are a severe but often ignored error source in RNAi experiments. This is particularly important for genome-wide screening approaches, in which only a number of hits can be validated individually (39–41). In fact, it has been reported that in an RNAi screen for cell cycle regulators, MAD2—a key protein in this process—was frequently off-targeted leading to great numbers of false positives (36). These observations underscore the urgent need of RNAi reagents without off-target effects.

The reduction of off-target effects has been a major direction in RNAi research since such effects had been recognized (41). A widely used strategy to reduce off-target effects is the introduction of chemical modifications into guide and passenger strands. 2′-*O*-methylation of the guide strand leads to a reduction of miRNA-like off-target effects presumably due to less efficient binding to the seed sequence of the off-target mRNA. For siRNA on-target activity, this modification seems to be tolerated (42). In addition to 2′-*O*-methyl groups, locked nucleic acids or deoxynucleotides have been used and off-target reduction was also observed (43,44). A systematic screen for suitable chemical modifications found that destabilizing unlocked nucleic acid modifications at position 7 of the guide strand resulted in significantly reduced off-target effects (45,46). Moreover, it has also been reported that the utilization of miRNA-like siRNAs, i.e. mismatched miRNA/miRNA* duplexes, possess potent on-target activity but approximately 10-fold reduced off-target effects (47,48). Since both the guide and the passenger strand can cause off-target effects, it has been demonstrated that passenger strand loading and thus all potential passenger strand mediated off-target effects can be reduced by using a fragmented passenger strand (referred to as sisiRNAs) or by blocking the 5′ end of the passenger strand by methylation (49,50). Taken together, numerous chemical modifications at many different positions of the guide as well as the passenger strand have been introduced (reviewed in (51)) and these alterations have significantly improved siRNA specificity and reduced miRNA-like off-target effects. However, despite all these improvements, particularly siRNA screens still generate high numbers of false positives mainly due to off-target effects (52) highlighting the need of further siRNA specificity improvements.

We present a next-generation RNAi tool that is based on complex pools of selected siRNA sequences, which we refer to as siPools. SiRNA mixtures are already used for RNAi applications. These pools are generated by RNase III or Dicer, which cleave the long dsRNAs in a stochastic process to all possible double-stranded cleavage products (53–55). In addition, RNase III, for example generates not exclusively 21 mers but also a large variety of small RNAs with different lengths. All these unspecific RNAs can cause additional and unpredictable problems in RNAi experiments. Of note, biochemical studies have demonstrated that a single point mutation in *Escherichia coli* RNase III generates more discrete size products thus improving RNase III generated siRNAs (56). On the other hand, defined pools containing four siRNAs are available (referred to as smart pools). However, because of the low complexity, off-target effects are not significantly reduced, while the on-target activity is not improved compared to single siRNAs. Higher pool complexities have not been used because mixing synthetic siRNAs is rather cost-intensive and therefore not realistic as a commercial tool. We solve this problem by an enzymatic siPool-production protocol, which is cheap and robust. SiPools combine the strengths of the two existing pooling approaches: first, our siPools are complex enough to dilute out the sequence-specific off-target effects and second, siPools are accurately defined allowing for the selection of highly potent siRNAs and target sequences leading to highly efficient knockdown results. It should be noted that siRNA pooling approaches might not be suitable for therapeutic purposes. Authorities might not approve even well-defined pools such as siPools because they resemble complex compound mixtures.

In addition to the major step forward in eliminating off-target effects, our siPools may have several other advantages. First, redundant gene family members can be efficiently targeted simultaneously. Smaller pools against each member can be generated, which can easily be combined to larger pools with wider but highly specific target spectra. Second, whole cellular pathways might be targeted with one siPool containing siRNAs against several key factors, which might be located at pathway branch points. Third, viral RNAs, which mutate rapidly and frequently escape siRNA targeting strategies (57), could be targeted with siPools much more efficiently. Finally, our siPool approach would also allow for inclusion of modified nucleotides, which could be selectively added to the *in vitro* transcription reaction of the passenger strand. This would lead to siRNAs with modified passenger strands only. Such modified siPools might be more stable when injected into animals, for example, and might therefore be ideal reagents for *in vivo* studies.

Recently, a large number of long non-coding RNAs (lncRNAs or lincRNAs) have been discovered in various organisms and tissues (58,59). These RNAs have different functions and are mainly involved in gene regulation at the chromatin level. LncRNAs can be several kilobases in length and are usually highly structured and packaged into RNA–protein complexes. Thus, not many regions might be accessible for knockdown experiments and it has been observed that such RNAs are notoriously difficult to knock down using single siRNAs. It is tempting to speculate that our siPools solve this problem since enough siRNAs within a complex mixture might find accessible binding sites on a lncRNA. Our own preliminary data support this hypothesis (data not shown). This might not only be possible for cytoplasmic lncRNAs since it has been shown that RNAi works well in the nucleus (60,61). In summary, siPools might therefore not only control off-target activity of siRNAs but could also serve as a potent tool to inactivate lncRNAs.

## ACCESSION NUMBERS

Raw intensities and normalized expression data of the microarrays are publicly available at the NCBI Gene Expression Omnibus (GEO, http://www.ncbi.nlm.nih.gov/geo/) under accession GSE57674.

## SUPPLEMENTARY DATA

Supplementary Data are available at NAR Online.

SUPPORTING INFORMATION
