# Supplementary material for: siPools: highly complex but accurately defined siRNA pools eliminate off-target effects
Source: Nucleic Acids Res. 2014 May 28;42(12):8049–61. doi: 10.1093/nar/gku480 (PMC4081087; doi:10.1093/nar/gku480)
Supplement: SUPPORTING INFORMATION [file supp_42_12_8049__index.html]

siPools: highly complex but accurately defined siRNA pools eliminate off-target effects — SUPPORTING INFORMATION 

# siPools: highly complex but accurately defined siRNA pools eliminate off-target effects

## SUPPORTING INFORMATION

**Files in this Data Supplement:**

- Supplemental Figure 1
- Supplemental Figure 2
- Supplemental Figure 3
- Supplemental Figure 4
- Supplemental Data
- Supplemental Table 1
