# Supplementary figures and images for: siPools: highly complex but accurately defined siRNA pools eliminate off-target effects
Source: Nucleic Acids Res. 2014 May 28;42(12):8049–61. doi: 10.1093/nar/gku480 (PMC4081087; doi:10.1093/nar/gku480)

**A**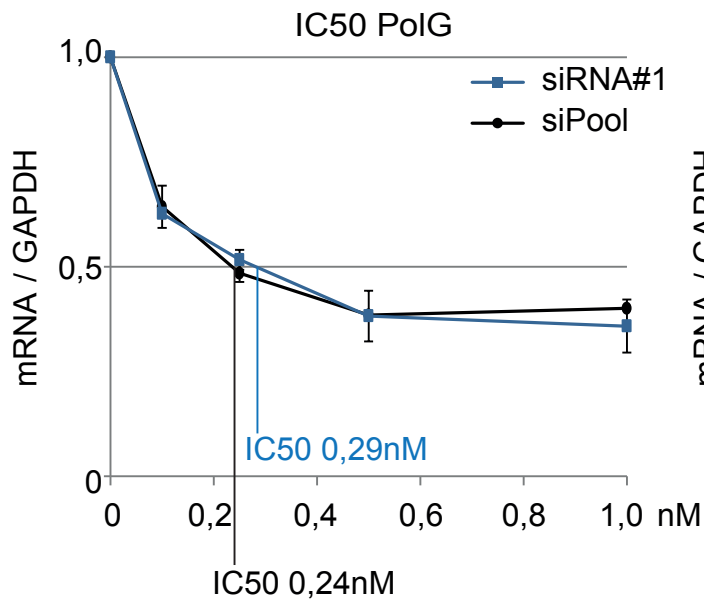**B**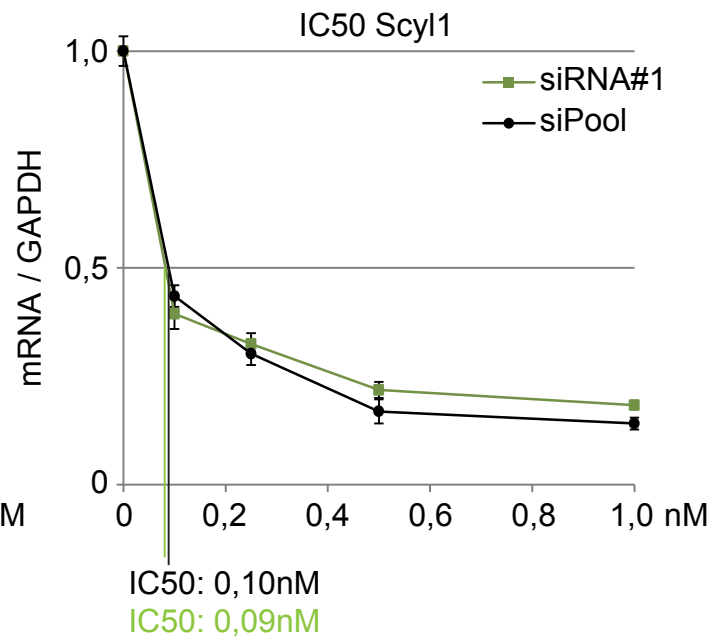

Supplement: SUPPORTING INFORMATION [file supp_gku480_nar-03360-y-2013-File008.pdf]

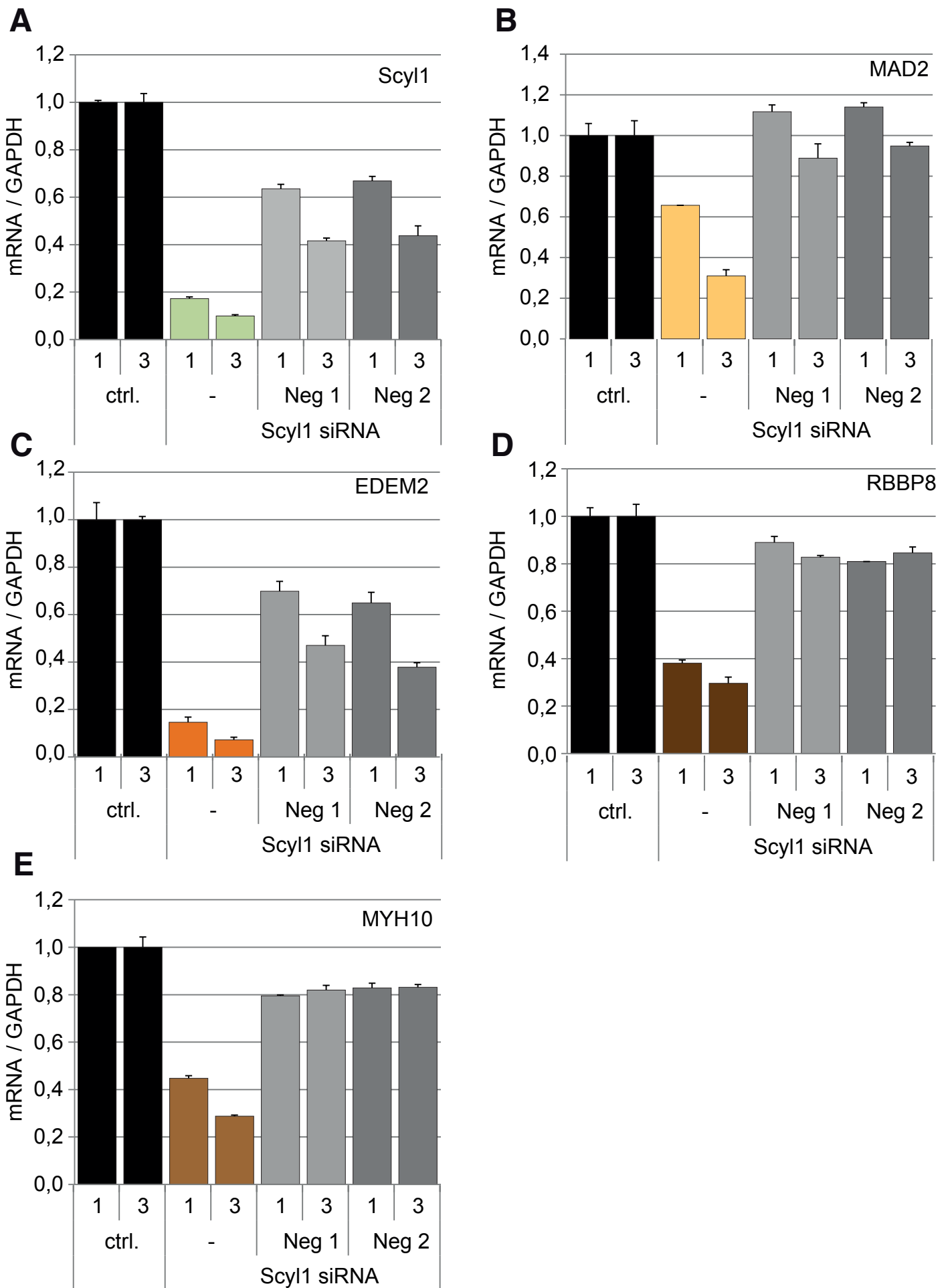

Supplement: SUPPORTING INFORMATION [file supp_gku480_nar-03360-y-2013-File009.pdf]

**A**
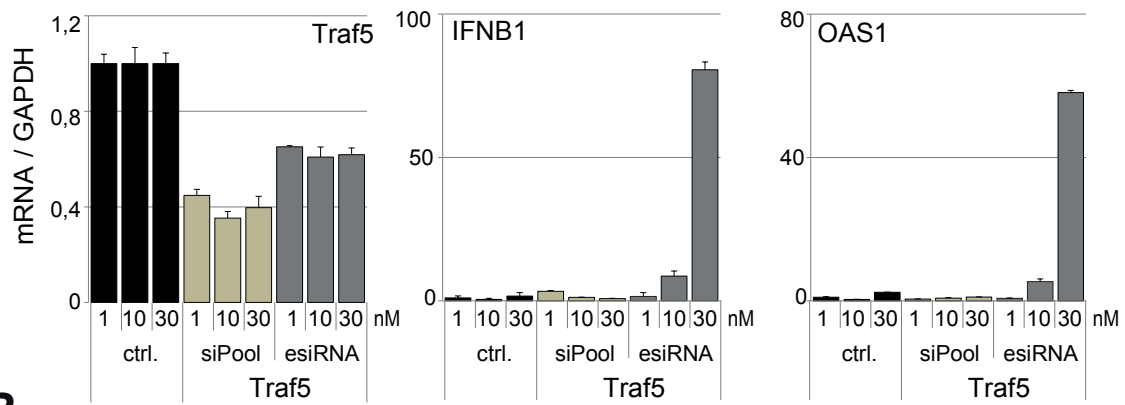
**B**
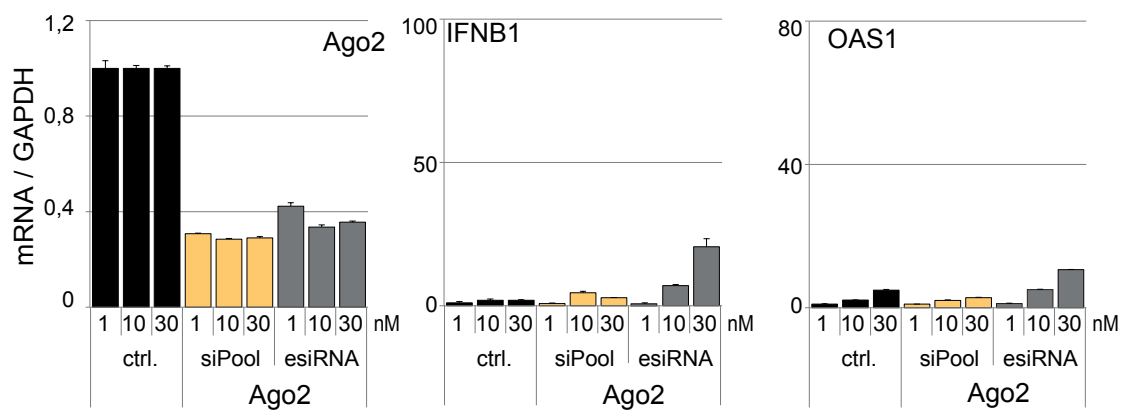

Supplement: SUPPORTING INFORMATION [file supp_gku480_nar-03360-y-2013-File010.pdf]

**A**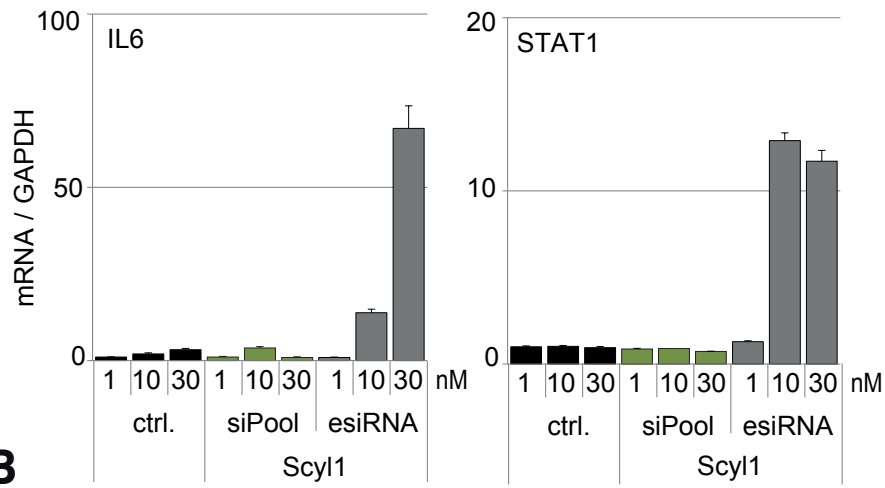**B**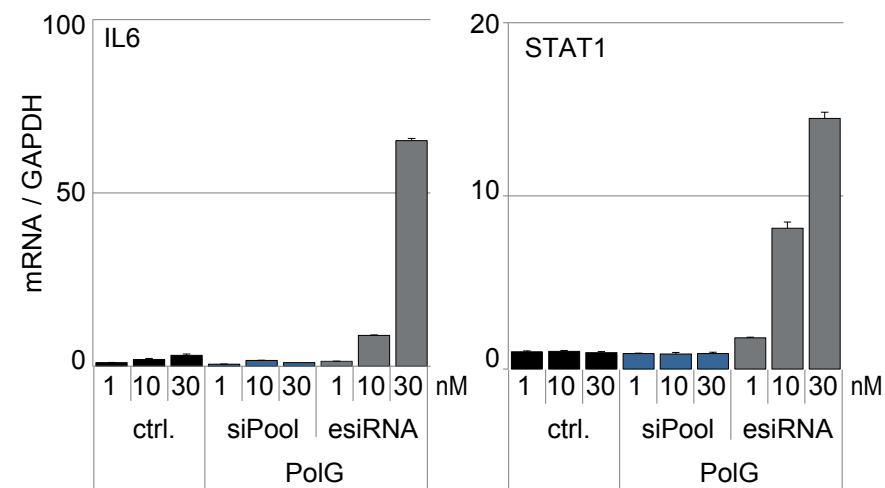**C**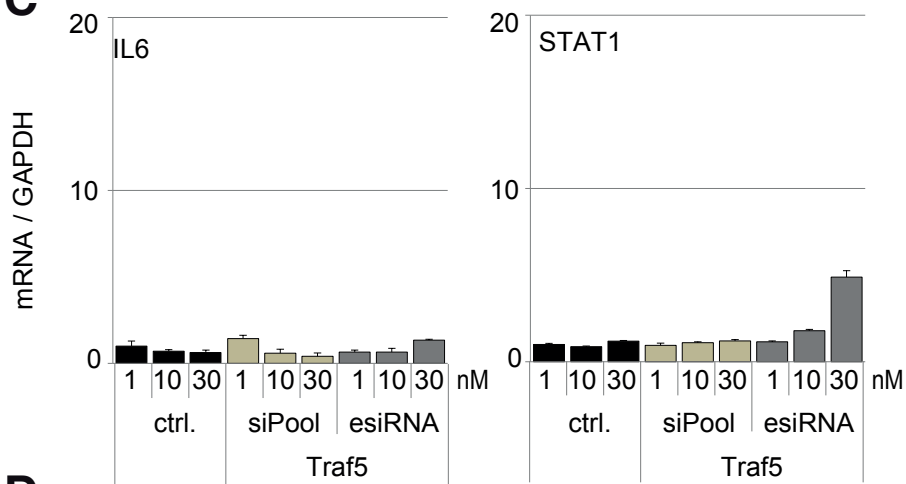**D**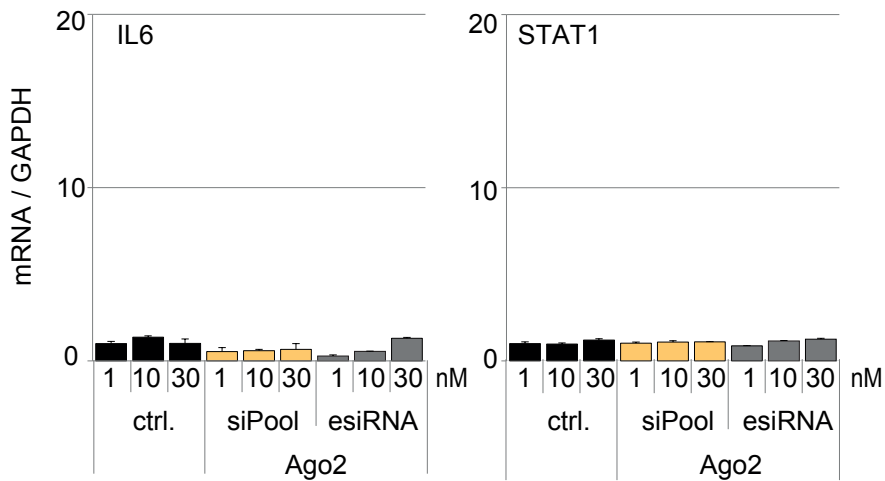

Supplement: SUPPORTING INFORMATION [file supp_gku480_nar-03360-y-2013-File011.pdf]
