# Supplementary material for: siPools: highly complex but accurately defined siRNA pools eliminate off-target effects
Source: Nucleic Acids Res. 2014 May 28;42(12):8049–61. doi: 10.1093/nar/gku480 (PMC4081087; doi:10.1093/nar/gku480)
Supplement: SUPPORTING INFORMATION [file supp_gku480_nar-03360-y-2013-File012.docx]

**siPools: highly complex but accurately defined siRNA pools eliminate off-target effects**

Michael Hannus^1,2^, Michaela Beitzinger^1^, Julia C. Engelmann^3^, Marie-Theresa Weickert^1^, Rainer Spang^3^, Stefan Hannus^2^ and Gunter Meister^1*^

**suppl. Material and Methods**

**siPool template sequences**

**PolG pool 1 sense**

GAATTCTAATACGACTCACTATAGGAGTTTGGCCCCAAAGTTCACATTAAAGTTTGGGAAAGAATTAATGCTCTAAGTTTGCTGATGCAGTGCCCTAGAAAGTTTGGGGTGAAGCGCTGGATATTAGTTTGCATTGTTGCTTGTTGGGTAAGTTTGGGTGTGGACTACAGGACAAAGTTTGGATAATTGAACTCACCAAAAGTTTGCAGGAAGAGTTTATGACCAAGTTTGGCTTACTAATGCAGTTTAAAGTTTGCCACAGAGCTCCTGCCCAAAGTTTGAGGAGGAGTTTCAACAAGAAGTTTGCTAAGAAGGTGAAGAAGGAAGTTTGGCCAGAAGTCCCAGAGGAAAGTTTGGCGTCGAGCACCTGCAGAAAGTTTGCGAGCAAATCTTCGGGCAAAGTTTGAAGCTT

**PolG pool 1 antisense**

GAATTCTAATACGACTCACTATAGGAGTTTGTTGCCCGAAGATTTGCTCGAGTTTGTTCTGCAGGTGCTCGACGCAGTTTGTTCCTCTGGGACTTCTGGCAGTTTGTCCTTCTTCACCTTCTTAGAGTTTGTCTTGTTGAAACTCCTCCTAGTTTGTTGGGCAGGAGCTCTGTGGAGTTTGTTAAACTGCATTAGTAAGCAGTTTGTGGTCATAAACTCTTCCTGAGTTTGTTTGGTGAGTTCAATTATCAGTTTGTTGTCCTGTAGTCCACACCAGTTTGTACCCAACAAGCAACAATGAGTTTGAATATCCAGCGCTTCACCCAGTTTGTTCTAGGGCACTGCATCAGAGTTTGTAGAGCATTAATTCTTTCCAGTTTGTTAATGTGAACTTTGGGGCAGTTTGAAGCTT

**PolG pool 2 sense**

GAATTCTAATACGACTCACTATAGGAGTTTGCGTGATAAACCTGCTCCAAAGTTTGAATTAATGCTCTAACGTGAAGTTTGGCTCTGATGCAGTGCCCTAAGTTTGGCAGAAGCCCCAAAGTTCAAGTTTGGAATTCAGTGGGTTCAGAAAGTTTGAAGATTCCTTCTAACTGAAAGTTTGCAAGGAAGTCACAGTGGAAAGTTTGCGGTAGAAGAACTGGATTAAGTTTGCCATATGGCAAACGGTAGAAGTTTGGAAGAAGGAACCAGCCACAAGTTTGGAGAGAGGTACAAAGAAGAAGTTTGCCATGAAGGACATTCGTGAAGTTTGGAGTCAGAAATGTTCAATAAGTTTGCCACAAAGCAAGGCCAGAAAGTTTGCGGCACAACCCATTGGACAAGTTTGAAGCTT

**PolG pool 2 anti-sense**

GAATTCTAATACGACTCACTATAGGAGTTTGTGTCCAATGGGTTGTGCCGAGTTTGTTCTGGCCTTGCTTTGTGGAGTTTGTATTGAACATTTCTGACTCAGTTTGTCACGAATGTCCTTCATGGAGTTTGTCTTCTTTGTACCTCTCTCAGTTTGTGTGGCTGGTTCCTTCTTCAGTTTGTCTACCGTTTGCCATATGGAGTTTGTAATCCAGTTCTTCTACCGAGTTTGTTCCACTGTGACTTCCTTGAGTTTGTTCAGTTAGAAGGAATCTTAGTTTGTTCTGAACCCACTGAATTCAGTTTGTGAACTTTGGGGCTTCTGCAGTTTGTAGGGCACTGCATCAGAGCAGTTTGTCACGTTAGAGCATTAATTAGTTTGTTGGAGCAGGTTTATCACGAGTTTGAAGCTT

**PolG pool 3 sense**

GAATTCTAATACGACTCACTATAGGAGTTTGAATGCTCTAACGTGATAAAAGTTTGGTTCACATTAACTCAGGCAAGTTTGGACTACAGGACAAGGGGCAAGTTTGGCAGTTGAATTCAGTGGGTAGTTTGAGAGAGAAACTGCAAGGAAAGTTTGTGCGCAAGGTCCAGAGAGAAGTTTGGGGCATCAGCCGTGAGCATAGTTTGGGTAATAGCTGTAATGTGGAGTTTGTAGAAGAACTGGATTACTTAGTTTGGCAGTGAGGAGGAGGAGTTAGTTTGAAGCTAAGAAGGTGAAGAAAGTTTGCAGGAGAGAGGTACAAAGAAGTTTGGAGATGAAGAAGTCGTTGAAGTTTGGTGAGAACTTCCAGGACCTAGTTTGGCAGAGGTGCACAGACTTTAGTTTGAAGCTT

**PolG pool 3 anti-sense**

GAATTCTAATACGACTCACTATAGGAGTTTGAAAGTCTGTGCACCTCTGCAGTTTGAGGTCCTGGAAGTTCTCACAGTTTGTCAACGACTTCTTCATCTCAGTTTGTCTTTGTACCTCTCTCCTGAGTTTGTTCTTCACCTTCTTAGCTTAGTTTGAACTCCTCCTCCTCACTGCAGTTTGAAGTAATCCAGTTCTTCTAAGTTTGCCACATTACAGCTATTACCAGTTTGATGCTCACGGCTGATGCCCAGTTTGTCTCTCTGGACCTTGCGCAAGTTTGTTCCTTGCAGTTTCTCTCTAGTTTGACCCACTGAATTCAACTGCAGTTTGTGCCCCTTGTCCTGTAGTCAGTTTGTGCCTGAGTTAATGTGAACAGTTTGTTTATCACGTTAGAGCATTAGTTTGAAGCTT.

**PolG pool 4 sense**

GAATTCTAATACGACTCACTATAGGAGTTTGTTAATGCTCTAACGTGATAAGTTTGCTAGAAGGGGAAAGAATTAAGTTTGCATTAACTCAGGCATTTCAAGTTTGGGGCATTGTTGCTTGTTGGAGTTTGGCTCCCAAACTCAGGCTTTAGTTTGGGAAGAAGTGGGAGGTGGTAGTTTGAGGAAGTCACAGTGGAAGAAGTTTGCGCAAGGTCCAGAGAGAAAAGTTTGCTGAGAAGGCCCAGCAGATAGTTTGCTTACAACGACGTGGACATAGTTTGATGGCAAACGGTAGAAGAAAGTTTGGTGAGGAGGAGGAGTTTCAAGTTTGAAGAAAGCTAAGAAGGTGAAGTTTGTGAAGAAGTCGTTGATGGAAGTTTGGAGAAGGAGCCTCGAGAACAGTTTGAAGCTT

**PolG pool 4 anti-sense**

GAATTCTAATACGACTCACTATAGGAGTTTGGTTCTCGAGGCTCCTTCTCAGTTTGTCCATCAACGACTTCTTCAAGTTTGTCACCTTCTTAGCTTTCTTAGTTTGTGAAACTCCTCCTCCTCACAGTTTGTTCTTCTACCGTTTGCCATAGTTTGATGTCCACGTCGTTGTAAGAGTTTGATCTGCTGGGCCTTCTCAGAGTTTGTTTCTCTCTGGACCTTGCGAGTTTGTCTTCCACTGTGACTTCCTAGTTTGACCACCTCCCACTTCTTCCAGTTTGAAAGCCTGAGTTTGGGAGCAGTTTGCCAACAAGCAACAATGCCCAGTTTGTGAAATGCCTGAGTTAATGAGTTTGTAATTCTTTCCCCTTCTAGAGTTTGTATCACGTTAGAGCATTAAAGTTTGAAGCTT

**Scyl1 pool 1 sense**

GAATTCTAATACGACTCACTATAGGAGTTTGGTCGACAGGTCAAGGCTGAAGTTTGGAGCCACAATAAATTCTATAGTTTGGCCATCTCACGTGTACATAAGTTTGGCCTCATCCACAACAATGTAGTTTGGACCACAAATCCTCCAAATAGTTTGGGAGAAGGATGTCCATGCAAGTTTGGGAGCTTCCTGTCCAAATTAGTTTGGCTCTGCGGTCTCACTGTAAGTTTGGGCTACAGGCCAAGGATGAAGTTTGGTACATGGCTTCCTGGACAAGTTTGGAGTATCAGCAGAAGATCAAGTTTGGAGGATTTCTGTCGGCACAAGTTTGGCAAGAGCCTGGACGCATTAGTTTGGCAGAGTGGTCAGAGAGAAAGTTTGCGTTGGGAATATACCTCAAAGTTTGAAGCTT

**Scyl1 pool 1 anti-sense**

gaattctaatacgactcactataggagtttgttgaggtatattcccaacgagtttgttctctctgaccactctgcagtttgaatgcgtccaggctcttgcagtttgtgtgccgacagaaatcctcagtttgtgatcttctgctgatactcagtttgtgtccaggaagccatgtacagtttgtcatccttggcctgtagccagtttgtacagtgagaccgcagagcagtttgaatttggacaggaagctccagtttgtgcatggacatccttctccagtttgatttggaggatttgtggtcagtttgacattgttgtggatgaggcagtttgtatgtacacgtgagatggcagtttgatagaatttattgtggctcagtttgtcagccttgacctgtcgacagtttgaagctt

**Scyl1 pool 2 sense**

GAATTCTAATACGACTCACTATAGGAGTTTGCATAATCAGAGCCACAATAAGTTTGCATCTCACGTGTACATAATAGTTTGCCGAGAGGAAGGTGGCCAAAGTTTGCGACTGGAGCAGCTGGGAAAGTTTGAGACGCAGGAGGAGGACAAAGTTTGTGGAGGAAGTGGAGAAGGAAGTTTGCACTGTAGATCCTGAGAAAAGTTTGTTGCACGGCTACAGGCCAAAGTTTGCAAAGCTGAACGAGGCCAAAGTTTGGAGGAGTATCAGCAGAAGAAGTTTGCAGCCCGCTTCCTGCAGAAAGTTTGGTGAGCTGGTGGGAGCAAAAGTTTGCAGCAGACATGTGGCGCTTAGTTTGGGCAGAGTGGTCAGAGAGAAGTTTGTCGATGGACTGGAGACAGAAGTTTGAAGCTT

**Scyl1 pool 2 anti-sense**

GAATTCTAATACGACTCACTATAGGAGTTTGTCTGTCTCCAGTCCATCGAAGTTTGTCTCTCTGACCACTCTGCCAGTTTGAAGCGCCACATGTCTGCTGAGTTTGTTTGCTCCCACCAGCTCACAGTTTGTTCTGCAGGAAGCGGGCTGAGTTTGTCTTCTGCTGATACTCCTCAGTTTGTTGGCCTCGTTCAGCTTTGAGTTTGTTGGCCTGTAGCCGTGCAAAGTTTGTTTCTCAGGATCTACAGTGAGTTTGTCCTTCTCCACTTCCTCCAAGTTTGTTGTCCTCCTCCTGCGTCTAGTTTGTTCCCAGCTGCTCCAGTCGAGTTTGTTGGCCACCTTCCTCTCGGAGTTTGATTATGTACACGTGAGATGAGTTTGTATTGTGGCTCTGATTATGAGTTTGAAGCTT

**Scyl1 pool 3 sense**

GAATTCTAATACGACTCACTATAGGAGTTTGGTACATAATCAGAGCCACAAGTTTGCCAGGCCATCTCACGTGTAAGTTTGCCAAGTGAGCCGTGCTAGTAGTTTGTGACAGATGGGACGACGAAAGTTTGCAGGAGGAGGACAAGGACAAGTTTGCTGAGAAATCCGTGCGAGAAGTTTGTCAATGTGGAGCTGATGAAAGTTTGCTGTGGTGGTCAAGATGTTAGTTTGAGGAGTATCAGCAGAAGATAGTTTGTCAAAGAGCCAGCCGAGAAAGTTTGGTGGCAGAGTGGTCAGAGAAGTTTGCCCTCAGCTTCCTGGTCAAAGTTTGTGGCTTACATCGATGGACTAGTTTGCTTCAAAACTCTACGGCACAGTTTGCCGTGTCCATCTTCGTCTAAGTTTGAAGCTT

**Scyl1 pool 3 anti-sense**

GAATTCTAATACGACTCACTATAGGAGTTTGTAGACGAAGATGGACACGGAGTTTGGTGCCGTAGAGTTTTGAAGAGTTTGAGTCCATCGATGTAAGCCAAGTTTGTTGACCAGGAAGCTGAGGGAGTTTGTCTCTGACCACTCTGCCACAGTTTGTTCTCGGCTGGCTCTTTGAAGTTTGATCTTCTGCTGATACTCCTAGTTTGAACATCTTGACCACCACAGAGTTTGTTCATCAGCTCCACATTGAAGTTTGTCTCGCACGGATTTCTCAGAGTTTGTGTCCTTGTCCTCCTCCTGAGTTTGTTCGTCGTCCCATCTGTCAAGTTTGACTAGCACGGCTCACTTGGAGTTTGTACACGTGAGATGGCCTGGAGTTTGTGTGGCTCTGATTATGTACAGTTTGAAGCTT

**Scyl1 pool 4 sense**

GAATTCTAATACGACTCACTATAGGAGTTTGTAATCAGAGCCACAATAAAAGTTTGAGGCCATCTCACGTGTACAAGTTTGGCCCCACAGATGTATTTATAGTTTGACAGATGGGACGACGAAGAAGTTTGGGACAAGGACACAGCAGAGAGTTTGAGGAAGTGGAGAAGGATGTAGTTTGCTGTAGATCCTGAGAAATCAGTTTGGGCTCCTACCTCAGTGCTAAGTTTGAGTATCAGCAGAAGATCATAGTTTGACGCATTCCCTGAGGATTTAGTTTGCTGGTGGCTTCATGAGCAAAGTTTGGCAGCCTCATCCACAACAAAGTTTGGCTACACCAGATCGTGAAAAGTTTGTGAAGGAGCTGGAGATCTCAGTTTGCATCGATGGACTGGAGACAAGTTTGAAGCTT

**Scyl1 pool 4 anti-sense**

GAATTCTAATACGACTCACTATAGGAGTTTGTGTCTCCAGTCCATCGATGAGTTTGGAGATCTCCAGCTCCTTCAAGTTTGTTTCACGATCTGGTGTAGCAGTTTGTTGTTGTGGATGAGGCTGCAGTTTGTTGCTCATGAAGCCACCAGAGTTTGAAATCCTCAGGGAATGCGTAGTTTGATGATCTTCTGCTGATACTAGTTTGTAGCACTGAGGTAGGAGCCAGTTTGGATTTCTCAGGATCTACAGAGTTTGACATCCTTCTCCACTTCCTAGTTTGCTCTGCTGTGTCCTTGTCCAGTTTGTCTTCGTCGTCCCATCTGTAGTTTGATAAATACATCTGTGGGGCAGTTTGTGTACACGTGAGATGGCCTAGTTTGTTTATTGTGGCTCTGATTAAGTTTGAAGCTT

**TNRC6A pool sense**

gaattctaatacgactcactataggagtttgtcttgcactaaatccctgcagtttgttcacgaggataccgaggcagtttgtgatttgttaaagcactgcagtttgttgtagaagacacaggtccagtttgtacattctgaagccaactcagtttgtgggaaccaagtccagtgcagtttgtttgctttcaggagcatccagtttgttgttctctaatactggccagtttgttcagattcacaagtttccagtttgtagtaccataagagcctccagtttgtagggcctgaacatttgtcagtttgtaacttgaaagttagtgccagtttgtactctgggagtttgctgcagtttgttcctccagaatttgcgccagtttgttgctaggcagtttgttccagtttgtgtggcagaaccctgatccagtttgtgtactaccatcgctctccagtttgtagcagcagaatcatcttcagtttgtattccttgaagtttctccagtttgttcgttccaaccggaaacgagtttgtgctgaagtaccattatccagtttgtgccgcagcaatgggttccagtttgttccaggcaatggcatatcagtttgtttctttgtatatggtggcagtttgttgccactcagacccttcgagtttgtttgtttccacctttcatcagtttgtgtcttgtaacattcctccagtttgtctttggaaatctgaggccagtttgttgtgctggaggctgctgcagtttgtcaagtttgagttcaagccagtttgaagctt

**TNRC6A pool anti-sense**

gaattctaatacgactcactataggagtttgggcttgaactcaaacttgaagtttggcagcagcctccagcacaaagtttgggcctcagatttccaaagaagtttgggaggaatgttacaagacaagtttggatgaaaggtggaaacaaaagtttgcgaagggtctgagtggcaaagtttggccaccatatacaaagaaaagtttggatatgccattgcctggaaagtttgggaacccattgctgcggcaagtttgggataatggtacttcagcaagtttgcgtttccggttggaacgaaagtttgggagaaacttcaaggaataagtttggaagatgattctgctgctaagtttgggagagcgatggtagtacaagtttgggatcagggttctgccacaagtttgggaacaaactgcctagcaaagtttgggcgcaaattctggaggaaagtttggcagcaaactcccagagtaagtttgggcactaactttcaagttaagtttggacaaatgttcaggccctaagtttgggaggctcttatggtactaagtttgggaaacttgtgaatctgaaagtttgggccagtattagagaacaaagtttgggatgctcctgaaagcaaaagtttggcactggacttggttcccaagtttggagttggcttcagaatgtaagtttgggacctgtgtcttctacaaagtttggcagtgctttaacaaatcaagtttggcctcggtatcctcgtgaaagtttggcagggatttagtgcaagaagtttgaagctt

**TNRC6B pool sense**

gaattctaatacgactcactataggagtttgtagatttggaatctgatgcagtttgtggtgttccagactcctccagtttgtgtgggaggaagcactgccagtttgttggcaagaggattcatccagtttgtttggaattaggtggacggagtttgttgttgaggagataactggagtttgtttggtttgaaggtctgggagtttgtgaagccagaaccatatccagtttgtaaagcgagactcggttccagtttgtgtggctacagagggcagcagtttgtttgtgcatattggcttccagtttgtggcaggtaaccagctatcagtttgttggtggagatttggcaggagtttgtacctttccatggcactccagtttgtgtggtgttatcccgcagcagtttgtgagggaagaattagacccagtttgtaatcagggaactttgctgagtttgttgtgtcctatgggatctgagtttgttgttggagagatgagtggagtttgtcgttctcaaggttgacccagtttgtggaatgtcagcagtgggcagtttgtagattcagatggaatgtcagtttgtatcggatcagggcagtgcagtttgtcccaacacacacatgtgcagtttgttcatcatcagtggcaaacagtttgttgtgccagaaagcggctgagtttgtcccacgggatctgactggagtttgtgaagcgccagcaagatcgagtttgtaaaggagcagggctgcccagtttgttgaatccgaccctcctccagtttgaagctt

**TNRC6B pool anti-sense**

gaattctaatacgactcactataggagtttgggaggagggtcggattcaaagtttggggcagccctgctcctttaagtttgcgatcttgctggcgcttcaagtttgccagtcagatcccgtgggaagtttgcagccgctttctggcacaaagtttggtttgccactgatgatgaaagtttggcacatgtgtgtgttgggaagtttggcactgccctgatccgataagtttggacattccatctgaatctaagtttggcccactgctgacattccaagtttggggtcaaccttgagaacgaagtttgccactcatctctccaacaaagtttgcagatcccataggacacaaagtttgcagcaaagttccctgattaagtttggggtctaattcttccctcaagtttggctgcgggataacaccacaagtttgggagtgccatggaaaggtaagtttgcctgccaaatctccaccaaagtttggatagctggttacctgccaagtttgggaagccaatatgcacaaaagtttggctgccctctgtagccacaagtttgggaaccgagtctcgctttaagtttgggatatggttctggcttcaagtttgcccagaccttcaaaccaaaagtttgccagttatctcctcaacaaagtttgccgtccacctaattccaaaagtttgggatgaatcctcttgccaaagtttgggcagtgcttcctcccacaagtttgggaggagtctggaacaccaagtttggcatcagattccaaatctaagtttgaagctt

**TNRC6C pool sense**

gaattctaatacgactcactataggagtttgtcatgtccaagtacaggccagtttgtcttgtggcagcaagtttcagtttgtgcgctgaaagacacattcagtttgtggcagtccatttgtctgcagtttgtattccttcagcactgcccagtttgttcaccaccaggctgtacgagtttgtagttccagaagatgccgcagtttgtcttgggtcaagatcattcagtttgtttcctttcagacctagggagtttgtgtcattccaaccagacccagtttgttctgtttgaccggtaccgagtttgttctatcccacatgtttacagtttgtgctgtgccattatccaccagtttgttgggaagcagcattattcagtttgtgcaggaggaggtgctttcagtttgtcatgatccaggcctcatcagtttgtactcttcaaggcctcctcagtttgtctttggagattggcgggcagtttgtgtgcgcgcaacttgctgcagtttgttcaccgacaagccaccggagtttgtccagggatagcaccatgcagtttgttctggattaaatcgtaccagtttgtttacctccactcttgaggagtttgtaaccctggaggtggcctcagtttgtttcgaagaacgagccagcagtttgtggaatgtgataagaggccagtttgttgagtcagattcaggtggagtttgtaccggaccacagcattgcagtttgtgtgcagagacttctgggcagtttgttcttcaccagcgaactcgagtttgaagctt

**TNRC6C pool anti-sense**

gaattctaatacgactcactataggagtttgcgagttcgctggtgaagaaagtttggcccagaagtctctgcacaagtttggcaatgctgtggtccggtaagtttgccacctgaatctgactcaaagtttgggcctcttatcacattccaagtttggctggctcgttcttcgaaaagtttggaggccacctccagggttaagtttgcctcaagagtggaggtaaaagtttgggtacgatttaatccagaaagtttggcatggtgctatccctggaagtttgccggtggcttgtcggtgaaagtttggcagcaagttgcgcgcacaagtttggcccgccaatctccaaagaagtttggaggaggccttgaagagtaagtttggatgaggcctggatcatgaagtttggaaagcacctcctcctgcaagtttggaataatgctgcttcccaaagtttgggtggataatggcacagcaagtttggtaaacatgtgggatagaaagtttgcggtaccggtcaaacagaaagtttggggtctggttggaatgacaagtttgccctaggtctgaaaggaaaagtttggaatgatcttgacccaagaagtttggcggcatcttctggaactaagtttgcgtacagcctggtggtgaaagtttggggcagtgctgaaggaataagtttggcagacaaatggactgccaagtttggaatgtgtctttcagcgcaagtttggaaacttgctgccacaagaagtttgggcctgtacttggacatgaagtttgaagctt.

**Unspecific ctrl-pool sense (Neg 1 sense)**

gaattctaatacgactcactataggagtttgtgtacgcgtctcgcgatttagtttgtatacgcggtacgatcgttagtttgttcgcgtaatagcgatcgtagtttgtcggcgtagtttcgacgatagtttgtcgcgtaaggttcgcgtatagtttgtcgcgattttagcgcgtatagtttgtcgcgtatatacgctacgtagtttgtttcgcgaacgcgcgtaatagtttgtcgtatcgtatcgtaccgtagtttgttatcgcgcgttatcgcgtagtttgtctcgtaggtacgcgatctagtttgtcgtactcgatagcgcaatagtttgtttgcgataccgtaacgctagtttgtgcgtaaggcatgtcgtatagtttgttatcggcagttcgccgttagtttgtagcgcgacatctatcgctagtttgtcgtcgtatcagcgcgtttagtttgtacgcgaaactgcgttcgtagtttgtcgacgatagctatcgcgtagtttgtcgcgtaatacgcgatcgtagtttgtcgcgataatgttacgcgtagtttgttaacgcgctacgcgtattagtttgtcgcgtataggtaacgcgtagtttgttacgcgatcacgtaacgtagtttgttatcgcgcgtcgcgtaatagtttgttacgtactagtgcgtactagtttgtatacgccggttgcgtagtagtttgttcgcgtgcatagcgtaatagtttgtacgcgacctaatcgcgatagtttgtcgtacgctgaacgcgtatagtttgaagctt

**Unspecific ctrl-pool anti-sense (Neg 1 anti-sense)**

gaattctaatacgactcactataggagtttgatacgcgttcagcgtacgaagtttgatcgcgattaggtcgcgtaagtttgattacgctatgcacgcgaaagtttgactacgcaaccggcgtataagtttgagtacgcactagtacgtaaagtttgattacgcgacgcgcgataaagtttgacgttacgtgatcgcgtaaagtttgacgcgttacctatacgcgaagtttgaatacgcgtagcgcgttaaagtttgacgcgtaacattatcgcgaagtttgacgatcgcgtattacgcgaagtttgacgcgatagctatcgtcgaagtttgacgaacgcagtttcgcgtaagtttgaaacgcgctgatacgacgaagtttgagcgatagatgtcgcgctaagtttgaacggcgaactgccgataaagtttgatacgacatgccttacgcaagtttgagcgttacggtatcgcaaaagtttgattgcgctatcgagtacgaagtttgagatcgcgtacctacgagaagtttgacgcgataacgcgcgataaagtttgacggtacgatacgatacgaagtttgattacgcgcgttcgcgaaaagtttgacgtagcgtatatacgcgaagtttgatacgcgctaaaatcgcgaagtttgatacgcgaaccttacgcgaagtttgatcgtcgaaactacgccgaagtttgacgatcgctattacgcgaaagtttgaacgatcgtaccgcgtataagtttgaaatcgcgagacgcgtacaagtttgaagctt

**Neg 2 sense**

gaattctaatacgactcactataggagttaggtccgctacgttaaaacgaagttagcttcgaccgtccgtttataagttagcgcgttactgattaacgtaagttagcgccgatattgtcgatttaagttaggtatccgggtttcgtttaaagttaggcgatagtgaaattcgttaagttagcgcgagtatactcgtattaagttagggtcggattgctatacgtaagttaggtaccggaatcaacgtaaaagttaggtgtcgactgttaaacgaaagttaggccggatatcgttcgttaaagttagctagtccgggctcgtaaaaagttagccgcgttttggtaatcgtaagttaggtcgcgttgaataacgaaaagttagcacgcgtatagcgaaattaagttagccgtcgattctttatacgaagttagcacgatcggcaattaacgaagttaggcgttcgtatgtattacgaagttagccgatacgttccgtataaaagttaggcgacgaaaacatcgaataagttagcatcgcgaacccgttataaagttaggcgcgaatcacatacgataagttagccgtacgaaagtatatcgaagttaggtcgtacggaataattcgaagttagctacgcgactatatcgataagttaggcgatcgaatttattcgaaagttaggcgtcgtagagtcgaataaagttagctcgacgataatacgataaagttagcttacgcgttacgataataagttaggcgcgatacaatcgataaaagttagaagcttgcggccgc

**Neg 2 anti-sense**

gaattctaatacgactcactataggagttagtttatcgattgtatcgcgcagttagtattatcgtaacgcgtaagagttagttatcgtattatcgtcgagagttagttattcgactctacgacgcagttagttcgaataaattcgatcgcagttagtatcgatatagtcgcgtagagttagtcgaattattccgtacgacagttagtcgatatactttcgtacggagttagtatcgtatgtgattcgcgcagttagttataacgggttcgcgatgagttagtattcgatgttttcgtcgcagttagtttatacggaacgtatcggagttagtcgtaatacatacgaacgcagttagtcgttaattgccgatcgtgagttagtcgtataaagaatcgacggagttagtaatttcgctatacgcgtgagttagtttcgttattcaacgcgacagttagtacgattaccaaaacgcggagttagttttacgagcccggactagagttagttaacgaacgatatccggcagttagttcgtttaacagtcgacacagttagtttacgttgattccggtacagttagtacgtatagcaatccgaccagttagtaatacgagtatactcgcgagttagtaacgaatttcactatcgcagttagttaaacgaaacccggatacagttagtaaatcgacaatatcggcgagttagtacgttaatcagtaacgcgagttagtataaacggacggtcgaagagttagtcgttttaacgtagcggacagttagaagcttgcggccgc

**siPool siRNA sequences**

**POLG pool 1 sense**

GCCCCAAAGTTCACATTAA

GGAAAGAATTAATGCTCTA

CTGATGCAGTGCCCTAGAA

GGGTGAAGCGCTGGATATT

CATTGTTGCTTGTTGGGTA

GGTGTGGACTACAGGACAA

GATAATTGAACTCACCAAA

CAGGAAGAGTTTATGACCA

GCTTACTAATGCAGTTTAA

CCACAGAGCTCCTGCCCAA

AGGAGGAGTTTCAACAAGA

CTAAGAAGGTGAAGAAGGA

GCCAGAAGTCCCAGAGGAA

GCGTCGAGCACCTGCAGAA

CGAGCAAATCTTCGGGCAA

**POLG pool 1 anti-sense**

TTGCCCGAAGATTTGCTCG

TTCTGCAGGTGCTCGACGC

TTCCTCTGGGACTTCTGGC

TCCTTCTTCACCTTCTTAG

TCTTGTTGAAACTCCTCCT

TTGGGCAGGAGCTCTGTGG

TTAAACTGCATTAGTAAGC

TGGTCATAAACTCTTCCTG

TTTGGTGAGTTCAATTATC

TTGTCCTGTAGTCCACACC

TACCCAACAAGCAACAATG

AATATCCAGCGCTTCACCC

TTCTAGGGCACTGCATCAG

TAGAGCATTAATTCTTTCC

TTAATGTGAACTTTGGGGC

**POLG pool 2 sense**

CGTGATAAACCTGCTCCAA

AATTAATGCTCTAACGTGA

GCTCTGATGCAGTGCCCTA

GCAGAAGCCCCAAAGTTCA

GAATTCAGTGGGTTCAGAA

AAGATTCCTTCTAACTGAA

CAAGGAAGTCACAGTGGAA

CGGTAGAAGAACTGGATTA

CCATATGGCAAACGGTAGA

GAAGAAGGAACCAGCCACA

GAGAGAGGTACAAAGAAGA

CCATGAAGGACATTCGTGA

GAGTCAGAAATGTTCAATA

CCACAAAGCAAGGCCAGAA

CGGCACAACCCATTGGACA

**POLG pool 2 anti-sense**

TGTCCAATGGGTTGTGCCG

TTCTGGCCTTGCTTTGTGG

TATTGAACATTTCTGACTC

TCACGAATGTCCTTCATGG

TCTTCTTTGTACCTCTCTC

TGTGGCTGGTTCCTTCTTC

TCTACCGTTTGCCATATGG

TAATCCAGTTCTTCTACCG

TTCCACTGTGACTTCCTTG

TTCAGTTAGAAGGAATCTT

TTCTGAACCCACTGAATTC

TGAACTTTGGGGCTTCTGC

TAGGGCACTGCATCAGAGC

TCACGTTAGAGCATTAATT

TTGGAGCAGGTTTATCACG

**POLG pool 3 sense**

AATGCTCTAACGTGATAAA

GTTCACATTAACTCAGGCA

GACTACAGGACAAGGGGCA

GCAGTTGAATTCAGTGGGT

AGAGAGAAACTGCAAGGAA

TGCGCAAGGTCCAGAGAGA

GGGCATCAGCCGTGAGCAT

GGTAATAGCTGTAATGTGG

TAGAAGAACTGGATTACTT

GCAGTGAGGAGGAGGAGTT

AAGCTAAGAAGGTGAAGAA

CAGGAGAGAGGTACAAAGA

GAGATGAAGAAGTCGTTGA

GTGAGAACTTCCAGGACCT

GCAGAGGTGCACAGACTTT

**POLG pool 3 anti-sense**

AAAGTCTGTGCACCTCTGC

AGGTCCTGGAAGTTCTCAC

TCAACGACTTCTTCATCTC

TCTTTGTACCTCTCTCCTG

TTCTTCACCTTCTTAGCTT

AACTCCTCCTCCTCACTGC

AAGTAATCCAGTTCTTCTA

CCACATTACAGCTATTACC

ATGCTCACGGCTGATGCCC

TCTCTCTGGACCTTGCGCA

TTCCTTGCAGTTTCTCTCT

ACCCACTGAATTCAACTGC

TGCCCCTTGTCCTGTAGTC

TGCCTGAGTTAATGTGAAC

TTTATCACGTTAGAGCATT

**POLG pool 4 sense**

TTAATGCTCTAACGTGATA

CTAGAAGGGGAAAGAATTA

CATTAACTCAGGCATTTCA

GGGCATTGTTGCTTGTTGG

GCTCCCAAACTCAGGCTTT

GGAAGAAGTGGGAGGTGGT

AGGAAGTCACAGTGGAAGA

CGCAAGGTCCAGAGAGAAA

CTGAGAAGGCCCAGCAGAT

CTTACAACGACGTGGACAT

ATGGCAAACGGTAGAAGAA

GTGAGGAGGAGGAGTTTCA

AAGAAAGCTAAGAAGGTGA

TGAAGAAGTCGTTGATGGA

GAGAAGGAGCCTCGAGAAC

**POLG pool 4 anti-sense**

GTTCTCGAGGCTCCTTCTC

TCCATCAACGACTTCTTCA

TCACCTTCTTAGCTTTCTT

TGAAACTCCTCCTCCTCAC

TTCTTCTACCGTTTGCCAT

ATGTCCACGTCGTTGTAAG

ATCTGCTGGGCCTTCTCAG

TTTCTCTCTGGACCTTGCG

TCTTCCACTGTGACTTCCT

ACCACCTCCCACTTCTTCC

AAAGCCTGAGTTTGGGAGC

CCAACAAGCAACAATGCCC

TGAAATGCCTGAGTTAATG

TAATTCTTTCCCCTTCTAG

TATCACGTTAGAGCATTAA

**SCYL1 pool 1 sense**

GTCGACAGGTCAAGGCTGA

GAGCCACAATAAATTCTAT

GCCATCTCACGTGTACATA

GCCTCATCCACAACAATGT

GACCACAAATCCTCCAAAT

GGAGAAGGATGTCCATGCA

GGAGCTTCCTGTCCAAATT

GCTCTGCGGTCTCACTGTA

GGCTACAGGCCAAGGATGA

GTACATGGCTTCCTGGACA

GAGTATCAGCAGAAGATCA

GAGGATTTCTGTCGGCACA

GCAAGAGCCTGGACGCATT

GCAGAGTGGTCAGAGAGAA

CGTTGGGAATATACCTCAA

**SCYL1 pool 1 anti-sense**

ttgaggtatattcccaacg

ttctctctgaccactctgc

aatgcgtccaggctcttgc

tgtgccgacagaaatcctc

tgatcttctgctgatactc

tgtccaggaagccatgtac

tcatccttggcctgtagcc

tacagtgagaccgcagagc

aatttggacaggaagctcc

tgcatggacatccttctcc

atttggaggatttgtggtc

acattgttgtggatgaggc

tatgtacacgtgagatggc

atagaatttattgtggctc

tcagccttgacctgtcgac

**SCYL1 pool 2 sense**

CATAATCAGAGCCACAATA

CATCTCACGTGTACATAAT

CCGAGAGGAAGGTGGCCAA

CGACTGGAGCAGCTGGGAA

AGACGCAGGAGGAGGACAA

TGGAGGAAGTGGAGAAGGA

CACTGTAGATCCTGAGAAA

TTGCACGGCTACAGGCCAA

CAAAGCTGAACGAGGCCAA

GAGGAGTATCAGCAGAAGA

CAGCCCGCTTCCTGCAGAA

GTGAGCTGGTGGGAGCAAA

CAGCAGACATGTGGCGCTT

GGCAGAGTGGTCAGAGAGA

TCGATGGACTGGAGACAGA

**SCYL1 pool 2 anti-sense**

TCTGTCTCCAGTCCATCGA

TCTCTCTGACCACTCTGCC

AAGCGCCACATGTCTGCTG

TTTGCTCCCACCAGCTCAC

TTCTGCAGGAAGCGGGCTG

TCTTCTGCTGATACTCCTC

TTGGCCTCGTTCAGCTTTG

TTGGCCTGTAGCCGTGCAA

TTTCTCAGGATCTACAGTG

TCCTTCTCCACTTCCTCCA

TTGTCCTCCTCCTGCGTCT

TTCCCAGCTGCTCCAGTCG

TTGGCCACCTTCCTCTCGG

ATTATGTACACGTGAGATG

TATTGTGGCTCTGATTATG

**SCYL1 pool 3 sense**

GTACATAATCAGAGCCACA

CCAGGCCATCTCACGTGTA

CCAAGTGAGCCGTGCTAGT

TGACAGATGGGACGACGAA

CAGGAGGAGGACAAGGACA

CTGAGAAATCCGTGCGAGA

TCAATGTGGAGCTGATGAA

CTGTGGTGGTCAAGATGTT

AGGAGTATCAGCAGAAGAT

TCAAAGAGCCAGCCGAGAA

GTGGCAGAGTGGTCAGAGA

CCCTCAGCTTCCTGGTCAA

TGGCTTACATCGATGGACT

CTTCAAAACTCTACGGCAC

CCGTGTCCATCTTCGTCTA

**SCYL1 pool 3 anti-sense**

TAGACGAAGATGGACACGG

GTGCCGTAGAGTTTTGAAG

AGTCCATCGATGTAAGCCA

TTGACCAGGAAGCTGAGGG

TCTCTGACCACTCTGCCAC

TTCTCGGCTGGCTCTTTGA

ATCTTCTGCTGATACTCCT

AACATCTTGACCACCACAG

TTCATCAGCTCCACATTGA

TCTCGCACGGATTTCTCAG

TGTCCTTGTCCTCCTCCTG

TTCGTCGTCCCATCTGTCA

ACTAGCACGGCTCACTTGG

TACACGTGAGATGGCCTGG

TGTGGCTCTGATTATGTAC

**SCYL1 pool 4 sense**

TAATCAGAGCCACAATAAA

AGGCCATCTCACGTGTACA

GCCCCACAGATGTATTTAT

ACAGATGGGACGACGAAGA

GGACAAGGACACAGCAGAG

AGGAAGTGGAGAAGGATGT

CTGTAGATCCTGAGAAATC

GGCTCCTACCTCAGTGCTA

AGTATCAGCAGAAGATCAT

ACGCATTCCCTGAGGATTT

CTGGTGGCTTCATGAGCAA

GCAGCCTCATCCACAACAA

GCTACACCAGATCGTGAAA

TGAAGGAGCTGGAGATCTC

CATCGATGGACTGGAGACA

**SCYL1 pool 4 anti-sense**

TGTCTCCAGTCCATCGATG

GAGATCTCCAGCTCCTTCA

TTTCACGATCTGGTGTAGC

TTGTTGTGGATGAGGCTGC

TTGCTCATGAAGCCACCAG

AAATCCTCAGGGAATGCGT

ATGATCTTCTGCTGATACT

TAGCACTGAGGTAGGAGCC

GATTTCTCAGGATCTACAG

ACATCCTTCTCCACTTCCT

CTCTGCTGTGTCCTTGTCC

TCTTCGTCGTCCCATCTGT

ATAAATACATCTGTGGGGC

TGTACACGTGAGATGGCCT

TTTATTGTGGCTCTGATTA

**TNRC6A pool sense**

tcttgcactaaatccctgc

ttcacgaggataccgaggc

tgatttgttaaagcactgc

ttgtagaagacacaggtcc

tacattctgaagccaactc

tgggaaccaagtccagtgc

tttgctttcaggagcatcc

ttgttctctaatactggcc

ttcagattcacaagtttcc

tagtaccataagagcctcc

tagggcctgaacatttgtc

taacttgaaagttagtgcc

tactctgggagtttgctgc

ttcctccagaatttgcgcc

ttgctaggcagtttgttcc

tgtggcagaaccctgatcc

tgtactaccatcgctctcc

tagcagcagaatcatcttc

tattccttgaagtttctcc

ttcgttccaaccggaaacg

tgctgaagtaccattatcc

tgccgcagcaatgggttcc

ttccaggcaatggcatatc

tttctttgtatatggtggc

ttgccactcagacccttcg

tttgtttccacctttcatc

tgtcttgtaacattcctcc

tctttggaaatctgaggcc

ttgtgctggaggctgctgc

tcaagtttgagttcaagcc

**TNRC6A pool anti-sense**

ggcttgaactcaaacttga

gcagcagcctccagcacaa

ggcctcagatttccaaaga

ggaggaatgttacaagaca

gatgaaaggtggaaacaaa

cgaagggtctgagtggcaa

gccaccatatacaaagaaa

gatatgccattgcctggaa

ggaacccattgctgcggca

ggataatggtacttcagca

cgtttccggttggaacgaa

ggagaaacttcaaggaata

gaagatgattctgctgcta

ggagagcgatggtagtaca

ggatcagggttctgccaca

ggaacaaactgcctagcaa

ggcgcaaattctggaggaa

gcagcaaactcccagagta

ggcactaactttcaagtta

gacaaatgttcaggcccta

ggaggctcttatggtacta

ggaaacttgtgaatctgaa

ggccagtattagagaacaa

ggatgctcctgaaagcaaa

gcactggacttggttccca

gagttggcttcagaatgta

ggacctgtgtcttctacaa

gcagtgctttaacaaatca

gcctcggtatcctcgtgaa

gcagggatttagtgcaaga

**TNRC6B pool sense**

tagatttggaatctgatgc

tggtgttccagactcctcc

tgtgggaggaagcactgcc

ttggcaagaggattcatcc

tttggaattaggtggacgg

ttgttgaggagataactgg

tttggtttgaaggtctggg

tgaagccagaaccatatcc

taaagcgagactcggttcc

tgtggctacagagggcagc

tttgtgcatattggcttcc

tggcaggtaaccagctatc

ttggtggagatttggcagg

tacctttccatggcactcc

tgtggtgttatcccgcagc

tgagggaagaattagaccc

taatcagggaactttgctg

ttgtgtcctatgggatctg

ttgttggagagatgagtgg

tcgttctcaaggttgaccc

tggaatgtcagcagtgggc

tagattcagatggaatgtc

tatcggatcagggcagtgc

tcccaacacacacatgtgc

ttcatcatcagtggcaaac

ttgtgccagaaagcggctg

tcccacgggatctgactgg

tgaagcgccagcaagatcg

taaaggagcagggctgccc

ttgaatccgaccctcctcc

**TNRC6B pool anti-sense**

ggaggagggtcggattcaa

gggcagccctgctccttta

cgatcttgctggcgcttca

ccagtcagatcccgtggga

cagccgctttctggcacaa

gtttgccactgatgatgaa

gcacatgtgtgtgttggga

gcactgccctgatccgata

gacattccatctgaatcta

gcccactgctgacattcca

gggtcaaccttgagaacga

ccactcatctctccaacaa

cagatcccataggacacaa

cagcaaagttccctgatta

gggtctaattcttccctca

gctgcgggataacaccaca

ggagtgccatggaaaggta

cctgccaaatctccaccaa

gatagctggttacctgcca

ggaagccaatatgcacaaa

gctgccctctgtagccaca

ggaaccgagtctcgcttta

ggatatggttctggcttca

cccagaccttcaaaccaaa

ccagttatctcctcaacaa

ccgtccacctaattccaaa

ggatgaatcctcttgccaa

ggcagtgcttcctcccaca

ggaggagtctggaacacca

gcatcagattccaaatcta

**TNRC6C pool sense**

tcatgtccaagtacaggcc

tcttgtggcagcaagtttc

tgcgctgaaagacacattc

tggcagtccatttgtctgc

tattccttcagcactgccc

ttcaccaccaggctgtacg

tagttccagaagatgccgc

tcttgggtcaagatcattc

tttcctttcagacctaggg

tgtcattccaaccagaccc

ttctgtttgaccggtaccg

ttctatcccacatgtttac

tgctgtgccattatccacc

ttgggaagcagcattattc

tgcaggaggaggtgctttc

tcatgatccaggcctcatc

tactcttcaaggcctcctc

tctttggagattggcgggc

tgtgcgcgcaacttgctgc

ttcaccgacaagccaccgg

tccagggatagcaccatgc

ttctggattaaatcgtacc

tttacctccactcttgagg

taaccctggaggtggcctc

tttcgaagaacgagccagc

tggaatgtgataagaggcc

ttgagtcagattcaggtgg

taccggaccacagcattgc

tgtgcagagacttctgggc

ttcttcaccagcgaactcg

**TNRC6C pool anti-sense**

cgagttcgctggtgaagaa

gcccagaagtctctgcaca

gcaatgctgtggtccggta

ccacctgaatctgactcaa

ggcctcttatcacattcca

gctggctcgttcttcgaaa

gaggccacctccagggtta

cctcaagagtggaggtaaa

ggtacgatttaatccagaa

gcatggtgctatccctgga

ccggtggcttgtcggtgaa

gcagcaagttgcgcgcaca

gcccgccaatctccaaaga

gaggaggccttgaagagta

gatgaggcctggatcatga

gaaagcacctcctcctgca

gaataatgctgcttcccaa

ggtggataatggcacagca

gtaaacatgtgggatagaa

cggtaccggtcaaacagaa

gggtctggttggaatgaca

ccctaggtctgaaaggaaa

gaatgatcttgacccaaga

gcggcatcttctggaacta

cgtacagcctggtggtgaa

gggcagtgctgaaggaata

gcagacaaatggactgcca

gaatgtgtctttcagcgca

gaaacttgctgccacaaga

ggcctgtacttggacatga

**Unspecific ctrl-pool sense (Neg 1 sense)**

tgtacgcgtctcgcgattt

tatacgcggtacgatcgtt

ttcgcgtaatagcgatcgt

tcggcgtagtttcgacgat

tcgcgtaaggttcgcgtat

tcgcgattttagcgcgtat

tcgcgtatatacgctacgt

tttcgcgaacgcgcgtaat

tcgtatcgtatcgtaccgt

ttatcgcgcgttatcgcgt

tctcgtaggtacgcgatct

tcgtactcgatagcgcaat

tttgcgataccgtaacgct

tgcgtaaggcatgtcgtat

ttatcggcagttcgccgtt

tagcgcgacatctatcgct

tcgtcgtatcagcgcgttt

tacgcgaaactgcgttcgt

tcgacgatagctatcgcgt

tcgcgtaatacgcgatcgt

tcgcgataatgttacgcgt

ttaacgcgctacgcgtatt

tcgcgtataggtaacgcgt

ttacgcgatcacgtaacgt

ttatcgcgcgtcgcgtaat

ttacgtactagtgcgtact

tatacgccggttgcgtagt

ttcgcgtgcatagcgtaat

tacgcgacctaatcgcgat

tcgtacgctgaacgcgtat

**Unspecific ctrl-pool anti-sense (Neg 1 anti-sense)**

atacgcgttcagcgtacga

atcgcgattaggtcgcgta

attacgctatgcacgcgaa

actacgcaaccggcgtata

agtacgcactagtacgtaa

attacgcgacgcgcgataa

acgttacgtgatcgcgtaa

acgcgttacctatacgcga

aatacgcgtagcgcgttaa

acgcgtaacattatcgcga

acgatcgcgtattacgcga

acgcgatagctatcgtcga

acgaacgcagtttcgcgta

aaacgcgctgatacgacga

agcgatagatgtcgcgcta

aacggcgaactgccgataa

atacgacatgccttacgca

agcgttacggtatcgcaaa

attgcgctatcgagtacga

agatcgcgtacctacgaga

acgcgataacgcgcgataa

acggtacgatacgatacga

attacgcgcgttcgcgaaa

acgtagcgtatatacgcga

atacgcgctaaaatcgcga

atacgcgaaccttacgcga

atcgtcgaaactacgccga

acgatcgctattacgcgaa

aacgatcgtaccgcgtata

aaatcgcgagacgcgtaca

**Neg 2 sense**

gtccgctacgttaaaacga

cttcgaccgtccgtttata

cgcgttactgattaacgta

cgccgatattgtcgattta

gtatccgggtttcgtttaa

gcgatagtgaaattcgtta

cgcgagtatactcgtatta

ggtcggattgctatacgta

gtaccggaatcaacgtaaa

gtgtcgactgttaaacgaa

gccggatatcgttcgttaa

ctagtccgggctcgtaaaa

ccgcgttttggtaatcgta

gtcgcgttgaataacgaaa

cacgcgtatagcgaaatta

ccgtcgattctttatacga

cacgatcggcaattaacga

gcgttcgtatgtattacga

ccgatacgttccgtataaa

gcgacgaaaacatcgaata

catcgcgaacccgttataa

gcgcgaatcacatacgata

ccgtacgaaagtatatcga

gtcgtacggaataattcga

ctacgcgactatatcgata

gcgatcgaatttattcgaa

gcgtcgtagagtcgaataa

ctcgacgataatacgataa

cttacgcgttacgataata

gcgcgatacaatcgataaa

**Neg 2 anti-sense**

tttatcgattgtatcgcgc

tattatcgtaacgcgtaag

ttatcgtattatcgtcgag

ttattcgactctacgacgc

ttcgaataaattcgatcgc

tatcgatatagtcgcgtag

tcgaattattccgtacgac

tcgatatactttcgtacgg

tatcgtatgtgattcgcgc

ttataacgggttcgcgatg

tattcgatgttttcgtcgc

tttatacggaacgtatcgg

tcgtaatacatacgaacgc

tcgttaattgccgatcgtg

tcgtataaagaatcgacgg

taatttcgctatacgcgtg

tttcgttattcaacgcgac

tacgattaccaaaacgcgg

ttttacgagcccggactag

ttaacgaacgatatccggc

ttcgtttaacagtcgacac

tttacgttgattccggtac

tacgtatagcaatccgacc

taatacgagtatactcgcg

taacgaatttcactatcgc

ttaaacgaaacccggatac

taaatcgacaatatcggcg

tacgttaatcagtaacgcg

tataaacggacggtcgaag

tcgttttaacgtagcggac

**Supplementary Figure legends**

Suppl. Figure 1: siPools show similar knock down efficiacy like siRNAs. Hela cells were transfected with 0, 0,1, 0,25, 0,5 or 1nM concentrations of siPools (black) or siRNAs (color) targeting PolG (A) or Scyl1 (B). IC50 values are depicted in each graph. mRNA levels were measured via qPCR and normalized to GAPDH.

Suppl. Figure 2: Hela cells were transfected with 1nM or 3nM concentrations of SCYL1 off-T siRNA alone or mixed in an unspecific control siPool to simulate off target activity of the Scyl1 siRNA in an unrelated siPool. The siRNA was diluted into two different control siPools (unspecific control: Neg 1 or Neg 2) to equal concentrations of individual siRNAs in the pool. mRNA levels of SCYL1 (A) and the off-targets MAD2 (B) or via micro array analysis identified off-targets EDEM2 (C), RBBP8 (C) or MYH10 (D) were measured via qPCR and normalized to GAPDH. Relative expression levels were calculated based on transfection of an unspecific control siPool or an unspecific control siRNA (ctrl.).

Suppl. Figure 3: Analysis of interferon response induction. (A, B) MCF7 cells were transfected with 1, 10 or 30 nM siPools or corresponding esiRNAs against Traf5 (A) or Ago2 (B). mRNA levels were normalized to GAPDH and relative expression levels were calculated using a negative control siPool.

Suppl. Figure 4: Induction of interferon response by esiRNAs. (A-D) MCF7 cells were transfected with 1, 10 or 30 nM siPool or corresponding esiRNAs. Interferon response was measured using IL6 (right) and STAT1 (left) mRNA expression levels after transfection with siPools or esiRNAs targeting Scy1 (A), PolG (B), Traf5 (C) or Ago2 (D). mRNA levels were normalized to GAPDH and relative expression levels were calculated using a negative control siPool.
